# A systematic review of obstructive sleep apnea in Indigenous peoples worldwide

**DOI:** 10.1093/sleepadvances/zpag080

**Published:** 2026-07-23

**Authors:** Shannon L Edmed, Daniel P Sullivan, Michelle Olaithe, Scott Coussens, Philip Terrill, Dwayne Mann, Danielle L Wilson, Wayne Williams, Danny J Eckert, Sheethal Kalmadka, Sylistah Gadam, Yaqoot Fatima

**Affiliations:** Child Health Research Centre, The University of Queensland, Brisbane, Queensland, Australia; The Australian Research Council Centre of Excellence for Children and Families over the Life Course, The University of Queensland, Brisbane, Queensland, Australia; Child Health Research Centre, The University of Queensland, Brisbane, Queensland, Australia; School of Applied Psychology, Griffith University, Brisbane, Queensland, Australia; Griffith Centre for Mental Health, Griffith University, Brisbane, Queensland, Australia; Thompson Institute, University of the Sunshine Coast, Sippy Downs, Queensland, Australia; School of Psychological Science, University of Western Australia, Perth, Western Australia, Australia; Adelaide University, School of Society and Culture, Adelaide, South Australia, Australia; Women’s and Children’s Health Network, WCH Campus, North Adelaide, South Australia, Australia; School of Electrical Engineering and Computer Science, The University of Queensland, Brisbane, Queensland, Australia; School of Electrical Engineering and Computer Science, The University of Queensland, Brisbane, Queensland, Australia; Thompson Institute, University of the Sunshine Coast, Sippy Downs, Queensland, Australia; School of Electrical Engineering and Computer Science, The University of Queensland, Brisbane, Queensland, Australia; Thompson Institute, University of the Sunshine Coast, Sippy Downs, Queensland, Australia; Toowoomba Regional Clinical Unit, Medical School, Faculty of Health, Medicine and Behavioral Sciences, The University of Queensland, Toowoomba, Queensland, Australia; Flinders Health and Medical Research Institute (FHMRI) Flagship Sleep Health Program, Flinders University, Bedford Park, South Australia, Australia; Child Health Research Centre, The University of Queensland, Brisbane, Queensland, Australia; The Australian Research Council Centre of Excellence for Children and Families over the Life Course, The University of Queensland, Brisbane, Queensland, Australia; Thompson Institute, University of the Sunshine Coast, Sippy Downs, Queensland, Australia; The Australian Research Council Centre of Excellence for Children and Families over the Life Course, The University of Queensland, Brisbane, Queensland, Australia; Thompson Institute, University of the Sunshine Coast, Sippy Downs, Queensland, Australia

**Keywords:** sleep apnea, obstructive, indigenous peoples, health services accessibility, continuous positive airway pressure, health disparities, first nations, aboriginal and Torres Strait islander, Māori, native American/Alaska native, sleep health equity, culturally responsive care, CPAP adherence, systematic review

## Abstract

**Study Objectives:**

Emerging evidence indicates that Indigenous peoples experience disproportionately high rates of obstructive sleep apnea (OSA). This systematic review aimed to examine OSA prevalence, contributing factors, and intervention or treatment outcomes among Indigenous peoples worldwide.

**Materials and Methods:**

We systematically searched PubMed, Embase, CINAHL, Cochrane CENTRAL, ATSI Health, Web of Science and PsycINFO (last searched June 30, 2025), and grey literature for original research involving Indigenous peoples (≥18 years) diagnosed with OSA via self-report or laboratory-based measures (e.g. polysomnography). Data extraction focused on study characteristics and key findings. Methodological quality was assessed using NIH tools, and cultural appropriateness was evaluated using the CREATE Aboriginal and Torres Strait Islander Quality Appraisal Tool. A narrative synthesis was conducted.

**Results:**

From 599 records, 41 studies from six countries were included. OSA prevalence among Indigenous populations varied widely (community-based samples, 6.5%-69%; referred samples, 85%–95%; self-reported diagnosis, 1.2%–8.4%; hospital audit diagnoses, 0.8%–11.4%), with only one study reporting a robust population-based estimate (~6.5% among Māori). Severe cases predominated in referred samples. Factors associated with OSA included male sex, age, BMI, neck circumference, craniofacial and geographic factors, and symptoms such as snoring. Treatment evidence was limited to CPAP, with low adherence.

**Conclusions:**

Findings suggests that OSA is common among Indigenous peoples across diverse settings. However, prevalence estimates are heterogeneous and limited by differences in study design, sampling frame, and diagnostic approaches, with most evidence based on referred clinical samples. Improving awareness and culturally responsive screening and management may improve outcomes. Sleep health among Indigenous peoples warrants a higher priority in research, policy and practice.

**Registration:**

The protocol for this systematic review was registered with Prospero (CRD42024466932).

## Graphical Abstract

**Graphical Abstract:**
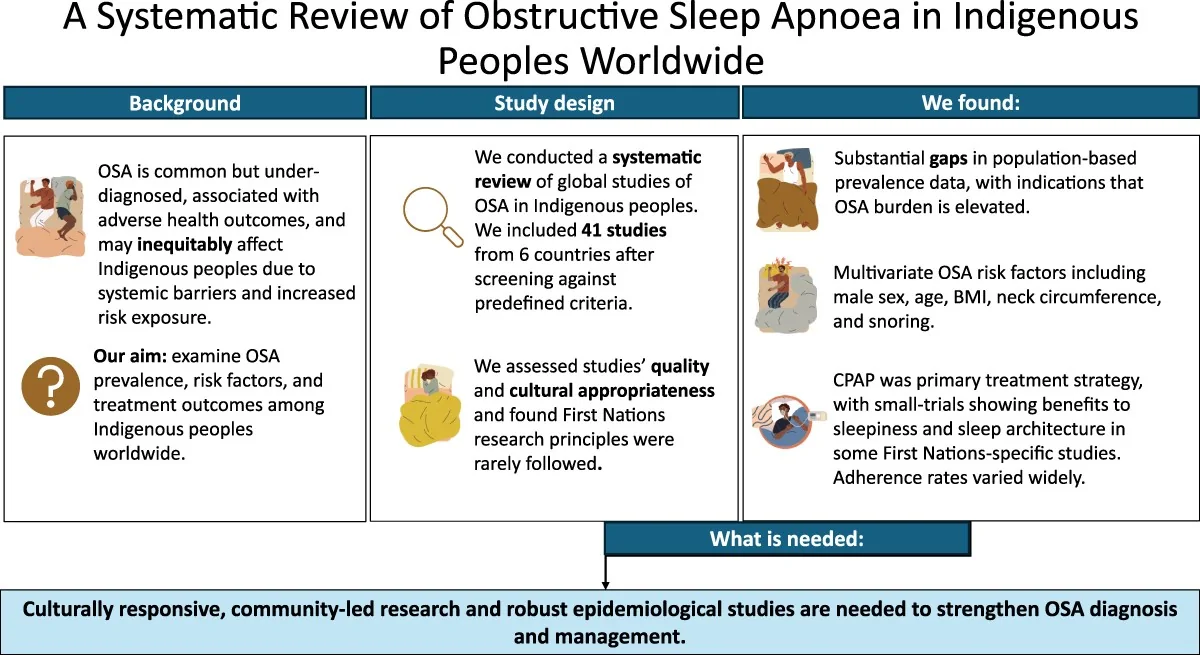

**Statement of Significance**

This systematic review provides a synthesis of evidence on obstructive sleep apnea prevalence, risk factors, and treatment outcomes among Indigenous peoples across six countries, incorporating both methodological quality and cultural appropriateness assessments of the included studies. Findings reveal substantial gaps in robust population-based data on the prevalence and determinants of obstructive sleep apnea, and limited evaluation of treatment effectiveness and adherence among Indigenous communities. These findings highlight the need for culturally responsive, community-led research to strengthen obstructive sleep apnea diagnosis and management.

## Introduction

Obstructive Sleep Apnea (OSA) is a prevalent but often underdiagnosed sleep disorder that adversely affects a range of health outcomes across many populations globally, including Indigenous and First Nations peoples.^1^ The prevalence of OSA in the general adult population is 9%-38% depending on the group measured, and even higher in some clinical populations [1–3]. A nocturnal breathing disorder, OSA is characterized by repeated episodes of partial or complete obstruction of the upper airway during sleep. These interruptions in breathing often lead to fragmented sleep and intermittent hypoxia, manifesting as symptoms such as loud snoring, gasping during sleep, and excessive daytime sleepiness.

Beyond the immediate impact of OSA on sleep quality and daytime function, OSA also contributes to an elevated risk of various chronic conditions, such as cardiovascular disease [2], type 2 diabetes [4], problems in memory and executive functioning [5, 6], anxiety and depression [7], and has been linked to increased rates of both industrial and motor vehicle accidents [8] and reduced quality of life [9]. While OSA adversely impacts all populations globally, its burden may be particularly pronounced among underserved and marginalized groups, including Indigenous peoples, who often face a range of systemic barriers to diagnosis and treatment, and may experience greater exposure to key risk factors [10].

In many countries, Indigenous peoples have been identified as priority populations or have dedicated health services and systems with a view to mitigate health disparities [11–13]. These health disparities are evident across a wide range of disease and health conditions including cardiometabolic syndromes, infectious diseases, conditions arising from environmental exposures (e.g. industrial pollution), and mental health disorders [14]. Sleep problems are no exception, with systematic reviews finding short sleep duration, fragmented and poor quality sleep, and excessive daytime sleepiness to be highly prevalent in First Nations Australian adults [10] and children [15].

Understanding OSA, and sleep more broadly, in the context of Indigenous peoples is critical from a health equity perspective. Disparities in sleep health, including the prevalence and impact of OSA, may illuminate broader patterns of health inequities experienced by Indigenous peoples globally [16]. Poor sleep health has been linked to a range of adverse outcomes, which can influence life opportunities, well-being, and long-term health trajectories [17, 18]. Conversely, good sleep health broadly supports physical, mental, and social outcomes [19]. By identifying the burden and determinants of OSA in Indigenous peoples, researchers and policymakers can pinpoint targets for intervention, participate in culturally appropriate healthcare strategies, and ultimately work toward reducing sleep-related and broader health disparities. Sleep health equity is therefore important to address immediate clinical needs but also a pathway to improve overall health outcomes and social equity for Indigenous peoples.

In Australia, First Nations peoples face an array of unique and multifaceted challenges in the diagnosis and management of OSA, highlighting that there remain critical gaps in our understanding of how best to support these communities [10, 20]. Barriers to timely diagnosis include: (1) low awareness of sleep disorders in general within First Nations communities [21]; (2) limited access to healthcare services, particularly in geographically remote areas [22, 23], and; (3) a lack of culturally appropriate health education and screening tools. These already serious factors are often compounded by broader systemic issues, including a high level of mistrust of mainstream health services, experiences of health-related stigma surrounding OSA, and the absence of culturally safe care environments, all of which can discourage engagement with the limited sleep health services that are available [21, 24].

Even following an appropriate diagnosis, OSA management among First Nations peoples may be compromised by limited access to follow-up care, as well as by a lack of tailored treatment approaches that are culturally responsive to community values and contexts [25]. These challenges are particularly amplified in regional and remote settings because of the distance to services [22]. However, proximity to services does not necessarily mitigate barriers in urban settings, where First Nations peoples may experience challenges related to limited cultural responsiveness and poor engagement with tertiary sleep services. Empirical research is needed to better characterize how barriers to OSA treatment and diagnosis vary by geographic context for First Nations peoples.

Furthermore, uptake and adherence to therapy, particularly to the first line treatment option continuous positive airway pressure (CPAP), has been reported as suboptimal in some First Nations contexts; however, both the extent of adherence and the underlying reasons for poor uptake and adherence remain inadequately characterized in First Nations populations [20]. This reflects an evidence gap, comprising limited quantitative data on CPAP adherence and a scarcity of research exploring the lived experiences of Indigenous and First Nations individuals with OSA. Better documentation and understanding of adherence patterns are essential to elucidate the social, cultural, and structural drivers of these outcomes and for developing more effective, culturally responsive models of care.

Prior to the current paper, there have been no systematic reviews that have examined OSA in Indigenous peoples worldwide. An earlier integrative review by Woods et al. [20] found comparatively higher rates of OSA in Indigenous peoples in high-income countries, and additionally, higher rates of moderate–severe OSA (apnea hypopnoea/respiratory disturbance index >15 events per hour). It is not fully understood why OSA prevalence and severity is elevated in Indigenous peoples. Ethnic differences in OSA risk factors, such as obesity and craniofacial structure, may have an impact on this variation [26]. However, the review by Woods [20] suggests that higher prevalence rates were likely a function of co-occurring factors including socioeconomic and healthcare disadvantage.

Overall, there is an urgent need to improve identification and management of OSA in Indigenous peoples, to ultimately improve sleep and other health outcomes. Sleep health disparities continue to be recognized as a priority research issue, and as such, it is timely to expand the previous review on OSA among Indigenous peoples [20]. While Woods and colleagues’ integrative review allowed inclusion of diverse methodologies, we have additionally included assessment of methodological quality of included studies in the current systematic review. Understanding the quality of this literature would provide important information to evaluate and synthesize findings as well as to guide future research. The purpose of this Systematic Review is to examine the prevalence of OSA among Indigenous peoples worldwide, as well as associated risk factors of OSA within Indigenous populations. Finally, this review seeks to examine the types and effectiveness of interventions and management options (if any) for Indigenous peoples globally.

## Materials and Methods

The protocol for this systematic review was registered with Prospero (CRD42024466932). This review was guided by the preferred reporting items for systematic review and meta analyses (PRISMA) [27].

### Eligibility criteria

We included studies meeting our inclusion and exclusion criteria (Table 1). All literature searches were limited to publications in English language with no date limitation.

**Table 1:** Inclusion and exclusion criteria for studies examining obstructive sleep apnea among Indigenous and First Nations populations worldwide

Parameter	Inclusion	Exclusion
Population	Adults aged 18+ years, any worldwide First Nation community, all countries	Study includes children <18 years and/or non-First Nations people AND study does not report disaggregated subgroup level data for those participants who do meet inclusion criteria
Exposures	Diagnosed with OSA including self-report of a clinical diagnosis, standardised questionnaire, or objective measures (including home or laboratory-based polysomnography).	Study addresses uncomplicated snoring only, or central sleep apnea only
Comparison	For intervention and epidemiologic studies reporting exposure to an intervention, any comparison group will be eligible, including but not limited to: treatment as usual, waitlist, active control, and placebo/sham conditions	Nil
Outcomes	OSA prevalence rates, predictors of OSA occurrence/severity, impact/outcome of OSA on other health parameters/comorbid conditions, interventions provided, intervention and treatment response/effectiveness	
Study type	Original research articles of the following study types: Cross-sectional studies Retrospective cohort studies Prospective cohort studies Randomised controlled trials	Case study/series Commentaries/Perspectives/Opinions/Editorials Blogs/Newspapers Narrative Reviews, Systematic Reviews and Meta-Analyses Qualitative studies with no quantitative findings reported Non-English language studies Abstract-only, no full-text available

OSA = Obstructive sleep apnea.

### Information sources

On September 20, 2023, the following databases were searched: PubMed, Embase, CINAHL, Cochrane CENTRAL, ATSI Health, Web of Science and PsycINFO. Grey literature searches were conducted in Google, Google Scholar, GreyNet, CADTH Grey Matters, OpenGrey, BASE, and MedNAR. These searches were replicated on 30 June 2025, using date limiters from September 2023 to ensure more recent articles were included.

### Search strategy

The key terms for the search strategies related to: (1) Indigenous peoples, and; (2) Obstructive Sleep Apnea. The search strategies were developed in consultation with a medical research librarian. Key terms and subject areas within each component were combined with the Boolean operator “OR” and all four components were combined with the Boolean operator “AND”. The final search strings for this review are detailed in Supplementary Materials A.

### Grey literature searches

#### Google

Google searches were conducted in April 2024 using advanced search features, limiting the search to specific domains (.org, .gov, .gov.au, .govt.nz, .gc.ca, and .gov.uk) to find PDF documents, and using search terms as specified in the protocol to capture government-related reports. The first 20 entries from each domain were retrieved, resulting in 120 entries. Then, a similarity check was performed to exclude duplicates (n = 5) from existing records in Covidence. The remaining 115 entries were reviewed, and only one entry (a preprint that leads to original article) met the inclusion criteria and was included for data extraction.

#### Google scholar

Google Scholar searches were conducted in April 2024 using search terms as specified in the protocol. The first 100 results were retrieved, and duplicates (n = 44) from existing records in the main database searches recorded in Covidence were excluded. The remaining results (n = 56) were reviewed. Of these, four articles and two theses met the inclusion criteria. It should be noted that two of the journal articles drew from the same dataset, as did the two theses. Due to this data duplication, only four unique datasets from the six relevant studies were included for data extraction in the final analysis.

#### Other databases

In addition to Google searches of grey literature, the following databases were searched in September 2023, as suggested by Paez and colleagues [28]. The strings “apnea” and “apnoea” returned no results on GreyNet or CADTH Grey Matters, and 43 results in OpenGrey (“apnea” = 41, “apnoea” = 2), all of which were excluded on title review for not including Indigenous terms. Searching “sleep apn” in BASE returned seven results, with no relevant articles located. Searching keywords “sleep” and “Indigenous” in MedNar returned 20 irrelevant results.

##### Selection process

Two researchers (DS and SE) independently reviewed titles and abstracts of all records. Conflicts identified were resolved via discussion, and with input from a third senior researcher (YF) when required. Three researchers (SE, DS, and SG) independently screened the full-text articles. Again, conflicts were resolved by discussion, and in cases of disagreement, the third reviewer made the final tie-breaking decision, and/or the senior researcher (YF) was consulted. When multiple publications reported data from a fully overlapping sample, only the most recent study was retained. Due to this overlapping criterion, one study was deleted.

### Data extraction

A data extraction form was used to extract basic data on study identifier (first author, year), study type, peer-reviewed/grey literature status, outcome measures, and sample details (number of participants, age, population groups, as appropriate). Specific fields were used to address the review questions, by mapping: (1) predictors of OSA in Indigenous peoples; (2) objective-measure prevalence rates of OSA in Indigenous peoples; (3) self-reported physician diagnosis prevalence rates of OSA in Indigenous peoples; (4) effects of sleep apnea on health parameters and other health problems; (5) treatments for OSA received by Indigenous peoples; (6) predictors of treatment response in Indigenous peoples, and; (7) treatment response/outcomes in Indigenous peoples. Data extraction was carried out by SE and verified by SK and DS.

Some studies included in this review modelled Indigenous identity as a predictor variable in analyses. These findings were extracted, but not synthesized in this review. Race and ethnicity are social and political constructs, not biological determinants of health; their use as predictor variables risks obscuring the structural conditions that produce sleep health inequities. This position aligns with strengths-based reporting frameworks, which hold that framing Indigeneity as a risk factor pathologizes identity rather than addressing the systems that generate disadvantage [29, 30]. Studies employing this analytical approach are retained in the evidence tables to maintain transparency regarding the available literature, but the associated findings are not reported in the results or carried into the discussion.

### Quality assessment

Papers were assessed using the quality assessment tools of the US National Institutes of Health for each respective study type (cohort, cross-sectional). There were no universal tools for assessing the cultural appropriateness and responsiveness of Indigenous and First Nations research worldwide. As such, we used the CREATE group’s Aboriginal and Torres Strait Islander Quality Appraisal Tool [31] to assess the cultural appropriateness of the research. Studies that did not provide enough information for quality assessment and analysis or did not fit within the framework requirements were assigned as “no ability to assess” or “not applicable”. Quality Assessment was conducted by DS and SG.

## Results

### Study inclusion

**Figure 1:**
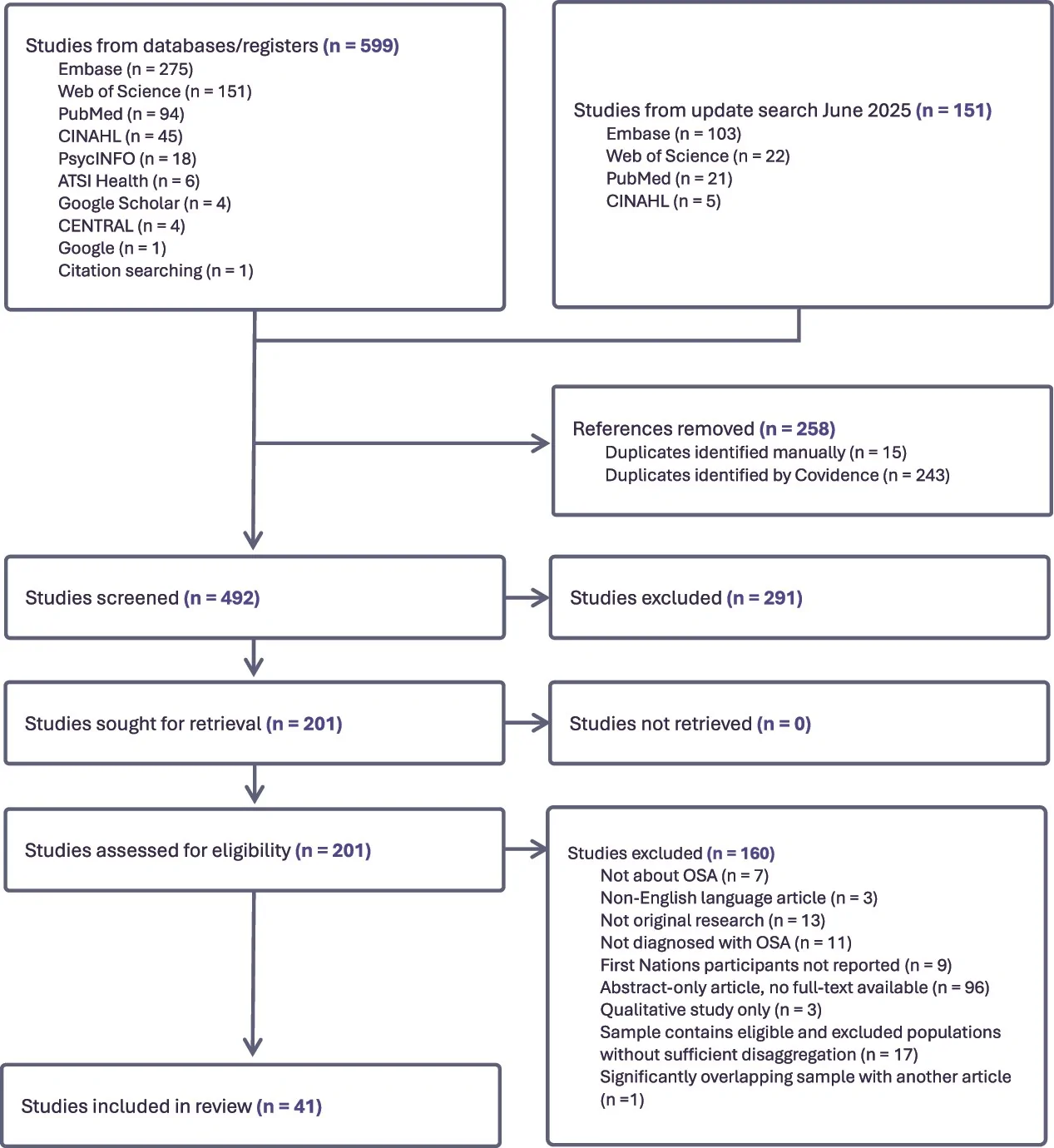
PRISMA flow diagram.

Figure 1 presents the flow of studies through the search and review process.

Due to heterogeneity among studies, a narrative synthesis was conducted.

### Overall characteristics of included studies

A total of 41 studies published between 1998 and 2024 were included [32–72]. Of these, 16 recruited participants from community settings [34–37, 40, 42, 44, 45, 50, 52, 55, 56, 65, 69, 70, 72], eight recruited participants from clinical or hospital settings [32, 33, 38, 39, 49, 59, 63], and 17 used data audits from clinics or hospitals [41, 43, 46–48, 51, 53, 54, 57, 60–62, 64, 66–68, 71]. One study recruited clients of the Machado-Joseph Disease Foundation [58].

The included studies represented Indigenous peoples from six countries. First Nations Australians (n = 15; 41, 43, 46–49, 51, 53, 57, 58, 62–64, 66), Māori from New Zealand (n = 5; 32, 33, 36, 38, 39), American Indian/Native-American, Native Alaskan, and Native Hawaiians/Pacific Islander peoples from the United States (n = 11; 34, 37, 54, 59, 60, 61, 67–69, 71, 72), First Nations peoples from Canada (n = 7; 35, 40, 50, 52, 55, 56, 65), and Amerindians from Ecuador (n = 3; 42, 44, 45). One study included participants from Samoa [70]. Half of the included studies focused exclusively on Indigenous peoples. These overarching study characteristics, along with the original aims of each article, are detailed in Supplementary Table S1.

### Prevalence of OSA across community-based, referred clinical samples, and audit-based studies

Table 2 summarizes sample-based prevalence of diagnosed OSA and, where available, severity categories. Table 2 groups studies by sampling frame to minimize the misinterpretation of prevalence estimates across multiple sources of prevalence estimates (e.g. community-based, referred clinical samples, special populations, and audit studies). Supplementary Table S2 reports the proportion of participants with OSA-related symptoms (e.g. snoring, witnessed apneas, and sleepiness) and those classified as “high risk” for OSA based on STOP-BANG screening.

**Table 2:** Prevalence of obstructive sleep apnea among indigenous populations across diverse settings and measurement methods grouped by sampling frame (community, self-reported community, referred clinical sample, special populations, and audit studies)

Study name (date), country (ref)	Indigenous population description; N/n (% First Nation of overall sample); % male; M/Mdn age (SD)	Recruitment setting; population context; exclusive versus diverse population groups	Measurement of OSA (PSG type/level); diagnostic threshold	OSA prevalence by study-specific criteria and severity
*Community samples*				
Mihaere (2009), New Zealand [36]	Māori, n = 3195(46%); 45% Male; M_age_ = NR. Subsample with overnight monitoring (n = 169)	Community-based recruitment; non-clinical setting; monitoring conducted in Maori and non-Maori participants.	Measurement of OSA: Level 4 PSG Index: RDI, 4% oxygen desaturations/h of sleep, plus bursts of snoring or ≥ 10/min increase in heart rate) Severity -Mild–moderate: RDI > 10 -Moderate–severe: RDI ≥ 15	Sample Prevalence % (N = 169) RDI > 10: 10.9% RDI ≥ 15: 6.5% *Sample prevalence of other groups:* Non-Maori (N = 195): RDI >10: 1.5%, RDI ≥ 15: 3.3%.
Punjabi (2009), United States [37]	Native American, n = 598 (9.5%); 47% Male; M_age_ = 62.9 (*SD* = 11).	Population-based recruitment from existing cohort studies; includes diverse racial groups; home sleep monitoring in a subsample for the Sleep Heart Health Study (which excludes those being treated for Sleep Disorder breathing).	Measurement of OSA: Level 2 PSG Total OSA: AHI ≥ 5 Severity (of those with OSA) -Mild: AHI 5-14.9 -Moderate: AHI 15-29.9 -Severe: AHI ≥ 30	Sample Prevalence % (n/N) Total: 55.8% (334/598) Mild: 60% Mod: 27% Severe: 12% *Sample prevalence of other groups: (*AHI ≥ 5 only)*:* White = 44.9%; African American = 44.2%; Hispanic = 39.3%; Other = 37.7%
Fonseca (2024), United States [69]	American Indians, N = 160 (100%); 30.63% Male; M_age_ = 62.6(SD = 5.4), with 139 participants undergoing PSG.	Population-based recruitment of American Indians from cohort studies (SleepHeart Health Study & Cerebrovascular Disease and its Consequences in American Indians (CDCAI) study); home sleep monitoring in a subsample.	Measurement of OSA: Level 2 PSG Total OSA: AHI ≥ 5 Severity (of those with OSA): -Mild: AHI 5–14 -Moderate: AHI 15–29 -Severe: AHI ≥30	Sample Prevalence % (n/N) Total: 78% (108/138) Mild: 40% Mod: 36% Severe: 23%
Wu (2024), United States [72]	American Indian participants, N = 123 (100%); 41% Male; M_age_ = 63.	Population-based recruitment of American Indians who were part of the Asthma Sub-study and the Sleep Heart Health Study; home sleep monitoring in a subsample.	Measurement of OSA: Level 2 PSG Total OSA: AHI ≥5.0 Severity (of those with OSA): -Mild REI of 5–15 -Moderate REI of 15–30 -Severe REI > 30	Sample Prevalence % (n/N) 10.5% (13/123) Mild: 69% Mod: 23% Severe: 8%
^^Del Brutto (2017), Ecuador [42]	Amerindian N = 38 (100%); 44% Male; M_age_ = 74.5(*SD* = 6.8).	Community-based recruitment; non-clinical setting; monitoring conducted exclusively among Amerindians.	Measurement of OSA: Level 1 PSG Total OSA: AHI ≥ 10 episodes per hour	Sample Prevalence % (n/N) Total: 39% (15/38)
^^Del Brutto (2019), Ecuador [44]	Amerindian N = 201 (100%); 36% Male; M_age_ = 71.2(*SD* = 7.5).	Community-based recruitment; non-clinical setting; monitoring conducted exclusively among Amerindians.	Measurement of OSA: Level 1 PSG Total OSA: AHI ≥ 5 Severity (of those with OSA): -AHI ≥5-14.9 -AHI ≥15	Sample Prevalence % (n/N) Total: 68% (137/201)AHI ≥5-14.9: 64%AHI ≥15: 36%
^^Del Brutto (2020), Ecuador [45]	Amerindian N = 167 (100%); 36% Male; M_age_ = 71.7(*SD* = 6.9).	Community-based recruitment; non-clinical setting; monitoring conducted exclusively among Amerindians.	Measurement of OSA: Level 1PSG Total OSA:AHI ≥ 5	Sample Prevalence % (n/N) Total: 68% (114/167) [75% (95/127) with positive neck grasp; 48% (19/40) with negative neck grasp].
Dosman (2022a), Canada [56]	Cree First Nations communities, N = 233(100%) 42% Male; M_age_ = 38(*SD* = 15).	Community-based recruitment; non-clinical setting; monitoring conducted exclusively among First Nation communities.	Measurement of OSA: Level 3 PSG Total OSA: AHI ≥ 5 Severity (of those with OSA): -AHI 5-14.9 -AHI 15-29.9 -AHI ≥ 30	Sample Prevalence % (n/N) Total: 45% (105/233) AHI 5-14.9: 73% AHI 15-29.9: 15% AHI ≥ 30: 11%
Heinsberg (2024), Samoa [70]	Samoan adults N = 330(100%); 46.7% Male; M_age_ = 52.9 (SD = 10).	Community-based recruitment, non-clinical setting; monitoring conducted exclusively among Samoan adults	Measurement of OSA: Level 4 PSG, categorical AHI 4%: Total OSA: ≥ 5 Severity (of those with OSA):Mild: 5 to >15 events/hr Moderate: 15 to <30 events/hr Severe: ≥30 events/hr	Sample Prevalence % (n/N) Total: 68% (226/330) Mild: 54% Mod: 31% Severe: 15%
*Community samples—Self reported diagnosis*				
Froese (2008), Canada [35]	Indigenous North American groups, N = 430 (100%); 44% Male; M_age_ = 43.2 (*SD* = 14.3).	Community-based recruitment; non-clinical setting; survey conducted exclusively among Indigenous North Americans.	Measurement of OSA: Self-reported physician-diagnosed condition (OSA)	Sample Prevalence % 1.2%
Pahwa (2015), Canada [40]	First Nation communities in Saskatchewan, N = 874 (100%); 49% male; M_age_ = 35.1(*SD* = 14.3).	Community-based recruitment; non-clinical setting; survey conducted exclusively among First Nation communities.	Measurement of OSA: Self-reported Sleep Apnea Diagnosis	Sample Prevalence % 8.3%
Singh (2020), Canada [50]	“Aboriginal” (e.g. First Nations, Métis, Inuit) n = 869 (6.1%); 32% Male; M_age_ = 43.5 (*SD* = 12.3).	Population-based recruitment; Canadian General Population; survey includes diverse racial groups.	Measurement of OSA: Self-reported physician-diagnosed condition (OSA)	Sample Prevalence % 8.4% *Sample prevalence of other groups:* Range = (2.4%-6.2%)
Karunanayake (2021), Canada [52]	Cree First Nations communities, N = 567(100%); 43.7% Male; M_age_ = 40(*SD* = 15.3).	Community-based recruitment; non-clinical setting; survey conducted exclusively among First Nation communities.	Measurement of OSA: Self-reported physician-diagnosed condition (OSA)	Sample Prevalence % 7%
Ikram (2023), Canada [65]	Cree First Nations communities, N = 588 (100%) 44.22% Male; 54.25% between 18-39 years.	Community-based recruitment; non-clinical setting; survey conducted exclusively among First Nation communities.	Measurement of OSA: Self-reported physician diagnosed sleep Apnea	Sample Prevalence % 6%
*Referred clinical samples*				
Baldwin (1998), New Zealand [32]	Māori, n = 48 (20.6%); 85% Male; *Mdn* Age = 48.5.	Sleep and respiratory clinic-based recruitment; referred for testing; includes diverse racial groups.	Measurement of OSA: Level 1 PSG Total OSA: apnea/ hypopnoea (A/H) index of >10 h-1 *and* excessive daytime sleepiness (ESS >11 or 2 answers indicating sleepiness).	Sample Prevalence % (n/N) Total: 85% (41/48) Sample prevalence of other groups: Pacific Islander = 91%; European = 49%
^**^Garg (2020), Australia [46]	“Indigenous Australians”, n = 60 (6.3%); % male: NR; Mdn age = 48.	Hospital/clinic-based medical records audit; includes Australians (including First Nations), referred for testing.	Measurement of OSA: Level 1 PSG Total OSA: AHI ≥ 5	Sample Prevalence % (n/N) Total: 95% (57/60)[46% Positional Sleep Apnea (POSA); 54% Non-POSA] *Sample prevalence of other groups:* Non-Indigenous = 84.9% (62% POSA; 38% Non-POSA)
^**^Heraganahally (2020), Australia [47]	Aboriginal and Torres Strait Islander People, n = 403(13%); 50.9% Male; Mdn age = 47.8.	Hospital/clinic-based medical records audit; Australians (including First Nations) referred for testing.	Measurement of OSA: Level 1 PSG Total OSA: AHI ≥ 5 Severity (of those with OSA): -Mild: AHI 5–15 -Moderate: AHI 15–30 -Severe: AHI >30	Sample Prevalence % (n/N) Total: 88.4% (350/396) Mild: 25.4% Mod: 22.6% Severe: 52% *Sample prevalence of other groups* (AHI ≥ 5 only): Non-ATSI = 83.0%
^**^Mehra (2020), Australia [48]	Aboriginal Australians^ diagnosed with OSA. N = 297 (100%) 51.8% Male; Mdn age = 47.	Specialist respiratory outreach service medical records audit; exclusively among Aboriginal Australians with OSA.	Measurement of OSA: Level 1 PSG Severity (of those with OSA): -Mild: AHI 5–15 -Moderate: AHI 15–30 -Severe: AHI >30	Sample Prevalence % (n/N) Mild: 25.6% (76/297) Mod: 22.2% (66/297) Severe: 52.2% (155/297)
^**^Heraganahally (2021), Australia [51]	Aboriginal Australians^ N = 340(100%); 51% Male; Mdn age = 47.	Hospital/clinic-based medical records audit; exclusively among Aboriginal Australians, between 2011 and 2015.	Measurement of OSA: Level 1 PSG Total OSA: AHI ≥ 5 Severity (of those with OSA): -Mild: AHI 5–15 -Moderate: AHI 15–30 -Severe: AHI >30	Sample Prevalence % (n/N) Total: 87% (297/340) Mild: 26% Mod: 23% Severe: 51%
^**^Heraganahally (2022), Australia [57]	“Indigenous Australians”, n = 86 (14.5%) 100% Women; Mdn age = 42.8.	Hospital/clinic-based medical records audit; includes Australian women (including First Nations), referred for testing, between 2014 and 2015.	Measurement of OSA: Level 1 PSG Total OSA: AHI ≥ 5 Severity (of those with OSA): -Mild: AHI 5–15 -Moderate: AHI 15–30 -Severe: AHI ≥30	Sample Prevalence % (n/N) Total: 85% (73/86) Mild: 33% Mod: 29% Severe: 38% *Sample prevalence of other groups: (*AHI ≥ 5 only): Non-indigenous = 76%
^**^Heraganahally (2023), Australia [62]	“Aboriginal Australians” (Aboriginal and/or Torres Strait Islander People), N = 730(100%), 649 with OSA (53% Male; Mdn age = 47.7.	Hospital/clinic-based medical records audit; exclusively among Aboriginal Australians with OSA, between 2011 to 2020	Measurement of OSA: Level 1 PSG Total OSA: AHI ≥ 5 Severity (of those with OSA): -Mild: AHI 5–15 -Moderate: AHI 15–30 -Severe: AHI >30	Sample Prevalence % (n/N) Total: 89% (649/730) Mild: 25% Mod: 25% Severe: 50%
^**^Heraganahally (2023), Australia [63]	“Indigenous Australians” N = 80 (100%); 51% Male; M_age_ = 45.1(SD = 11.54)	Sleep and respiratory clinic-based recruitment; referred for testing; exclusively among Indigenous Australians.	Measurement of OSA: Level 1 PSG Total OSA: AHI ≥ 5 Severity (of those with OSA): -Mild: AHI 5–15 -Moderate: AHI 15–30 -Severe: AHI ≥30	Sample Prevalence % (n/N) Total: 88% (70/80) Mild: 21% Mod: 21% Severe: 58%
^**^Howarth (2023), Australia [64]	“Aboriginal Australians”, N = 248 (100%) 48% Male; Mdn age = 46	Hospital/clinic-based medical records audit; exclusively among Aboriginal and/or Torres Strait Islander Australians, between 2012-2020.	Measurement of OSA: Level 1 PSG Total OSA: AHI ≥ 5 Severity (of those with OSA): -Mild: AHI 5–15 -Moderate: AHI 15–30 -Severe: AHI >30	Sample Prevalence % (n/N) Total: 89% (221/248) Mild: 26% Mod: 23% Severe: 51%
^**^Lindfield (2023), Australia [66]	“Aboriginal Australians” with OSA, N = 149 (100%) 54% Male; Mdn age = 48.89.	Retrospective cohort study (medical records audit), recruited from services within the Top End Health Service region between 2012-2020	Measurement of OSA: Level 1 PSG Severity (of those with OSA): -Mild: AHI 5–15 -Moderate:AHI 15–30 -Severe: AHI >30	Sample Prevalence % (n/N) Mild: 6% (9/149) Mod: 26% (38/149) Severe: 68% (102/149)
Locke (2023), United States [67]	Native Hawaiians/Pacific Islanders with OSA, N = 141 (100%) 68.7% Male; M_age_ = 46(*SD* = 13).	Medical records and administrative data audit from University of Utah Sleep Program. Native Hawaiians/Pacific Islanders with OSA.	Measurement of OSA: Level 1 and Level 3^#^ PSG Total OSA:AHI ≥ 5 Severity (of those with OSA): -“Mild” -“Moderate” -“Severe”	Sample Prevalence % (n/N) Total: 99.3% (140/141) Mild: 18% Mod: 24% Severe: 57%
*Special populations*				
Redhead (2020), Australia [49]	Aboriginal and Torres Strait Islander Women ≥26 weeks pregnant, n = 10 (8.8%); 100% women; M_age_ = NR	Hospital-based recruitment; includes women ≥26 weeks pregnant and diverse racial groups; home based monitoring.	Measurement of OSA: Level 4 PSG Total OSA: AHI ≥ 5	Sample Prevalence % (n/N) Total: 40% (4/10) *Sample prevalence of other groups:* Non-First Nations Australian (i.e. all other recorded ethnicities) = 25% (30/119); Caucasian = 13% (9/70); Pacific Islander = 33% (2/6).
^**^LaGrappe (2022), Australia [58]	Aboriginal and Torres Strait Islander Australians, N = 24 (100%); 29% Male; M_age_ = 45.6.	Convenience sampling from MJD Foundation clients; exclusively with First Nations Australians with Machado-Joseph Disease (MJD).	Measurement of OSA: Level 1/2 PSG Total OSA: AHI criteria (SDB/OSA if the total AHI was >5/hour)	Sample Prevalence % (n/N) Total: 47% (10/21)
*Audit Studies*				
^**^ Kruavit (2017), Australia [43]	“Indigenous Australians”, n = 352 (79.2%); 42.3% Male; M_age_ = 53.8(*SD* = 13.7).	Specialist respiratory outreach service medical records audit; Australians (including First Nations) referred for assessment.	Measurement of OSA: Clinical diagnosis of OSA as primary reason for referral, or as secondary diagnosis (co-existing condition). Primary diagnosis only Secondary diagnosis only	Sample Prevalence % Primary or secondary diagnosis: 11.4% Primary diagnosis: 8.2% Secondary diagnosis: 3.1% *Sample prevalence of other groups:* (primary or secondary diagnosis): Non-Indigenous = 41.3%
Naing (2021), Australia [53]	Aboriginal and Torres Strait Islander People, n = 791(45%); % Male: NR; M_age_ = 48(*SD* = 18).	Hospital/clinic-based medical records audit; including Australians with pulmonary hypertension (including First Nations).	Measurement of OSA: Clinical evidence of moderate to severe OSA comorbidity	Sample Prevalence % (n/N) 7% (56/789) *Sample prevalence of other groups:* Non-Indigenous = 15% (140/964)
Sanchez (2021), United States [54]	American Indian, n = 105 822(0.62%); 44.6% Male; M_age_ = 45.5(SD = 17.8).	Hospital/clinic-based medical records audit; diverse racial groups.	Measurement of OSA: Clinical diagnosis of OSA from medical records	Sample Prevalence % (n/N) 0.8% (847/105822) *Sample prevalence of other groups:* White = 1%; Black = 0.8%; Hispanic = 0.5%; Asian = 0.5%
Perkins (2022), United States [59]	American Indias/Alaska Natives (AI/AN) n = 43(46%) 48.8% Male; M_age_ = 51.9(SD = 12.7).	Hospital-based recruitment from ICU and non-ICU settings of patients who had SARS-CoV-2; includes diverse racial groups.	Measurement of OSA: Medical chart review and verbal interview (diagnosis)	Sample Prevalence % (n/N) 9.3% (4/43) *Sample prevalence of other groups:* Non AI/AN = 15.7%
Patel (2023), United States [68]	Native Americans N = 26 333 (0.5%) % male = NR Age = NR	Hospital-based medical records audit; stroke survivors from diverse racial groups.	Measurement of OSA: OSA as secondary diagnosis (based on ICD codes)	Sample Prevalence % (n/N) 3.24% (853/26333) *Sample prevalence of other groups:* Caucasian = 3.43%; African Americans = 3.27%; Hispanics = 2.42%; Asian or Pacific Islander = 1.6%

a) Prevalence estimates reflect the proportion of participants meeting study-specific diagnostic criteria for OSA. Comparative prevalence data for non-Indigenous or other ethnic groups are provided where available. Studies are presented in groups by sampling frame to distinguish between community-based prevalence estimates and referred/clinical or other non-population-based samples. b) All sleep studies were acquired from a single night of recording. Level 2-4 sleep studies were performed at home except where otherwise noted.

Studies marked ^**^originate from the same clinical service; studies marked ^^derive from the same cohort.

^ Does not include Torres Strait Islander or both Aboriginal and Torres Strait Islander identities.

^#^ Uncertain PSG level.

^##^ Semi-attended PSG outside of clinical environment (hotel accommodation). Regular observations conducted by an on-site nurse with on-call sleep technology and medical staff available.

Abbreviations: AHI = Apnea-Hypopnea index (events/hour); AI/AN = American Indian/Alaska Natives; ESS = Epworth Sleepiness Scale; ICU = Intensive Care Unity; MJD = Machado-Joseph Disease; Mdn = Median; M = Mean; N/n – Sample size/subsample size; NR = Not reported; PSG = Polysomnography; RDI = Respiratory Disturbance Index; REI = Respiratory Event Index; SD = Standard Deviation.

#### Community-based samples

Community-based studies using objective sleep assessment (e.g. PSG or home sleep testing) reported variable OSA prevalence estimates and severity. In a nationwide New Zealand survey subsample, Maori participants had higher prevalence than non-Maori at both RDI ≥ 15 events/h (6.5% vs 1.5%) and RDI ≥ 10 events/h (10.9% vs 3.3%) [36], approximating population-based estimates.

In contrast, studies of specific Indigenous communities reported higher prevalence: 45% among First Nations Canadians (AHI ≥ 5 events/h) [56], 69% among Samoan adults (AHI ≥ 5 events/h; AHI4% criterion) [70], and 39% (AHI ≥ 10 events/h) to 68% (AHI ≥ 5 events/h) in Ecuadorian Amerindian cohorts [42, 44, 45]. These studies used convenience sampling and should not be interpreted as population-level estimates or representative of broader populations.

In large U.S. cohort studies, estimates varied depending on the overlapping subsample selected. In a paper using data from the Sleep Heart Health Study, the derived prevalence was 55% (using AHI ≥ 5 events/h; sample prevalence for non-Indigenous participants were 45% among White participants, 39% among Hispanic participants, and 38% among participants of other racial and ethnic groups) [37], while overlapping subsamples ranged from 78% in the Cerebrovascular Disease and its Consequences in American Indian (CDCAI) study to 10.5% in the Asthma Sub-study [72], demonstrating the influence of selection bias.

Severity distributions in community-based studies, where available, typically showed a predominance of mild OSA, followed by moderate and then severe cases. This pattern contrasts with referred clinical samples (see below), where severe OSA was more common, suggesting that the most symptomatic individuals are more likely to be recognized and referred for sleep testing.

Community surveys relying on self-reported physician diagnosis revealed much lower prevalence estimates, ranging between 1.2% and 8.4% among Indigenous populations [35, 40, 50, 52, 65] in the United States and Canada. These figures likely reflect underdiagnosis and systematic access to sleep testing disparities, and findings may be influenced by participation bias and eligibility criteria.

#### Referred clinical samples and special populations

Among referred clinical samples assessed via in-laboratory or at-home PSG, sample-based prevalence of OSA (defined as AHI ≥ 5 events/h) was consistently high in Indigenous populations. For First Nations Australians, prevalence among referred samples ranged from 85% and 95% across studies [46, 47, 51, 57, 62–64], compared with 76%–84.9% in non-Indigenous peers [46, 47, 57]. For Māori and Pacific Islanders patients, prevalence among referred samples was 85% and 91% respectively, while prevalence was 49% for New Zealanders of European descent [32].

Severe OSA (AHI >30 events/h) was frequently observed among those diagnosed, affecting 50%–68% of mixed gender First Nations Australian samples [46–48, 51, 62–64, 66], 38% of First Nations women [57], and 57% of Native Hawaiians/Pacific islander patients [67]. These findings suggest a high burden of severe OSA among Indigenous patients referred for sleep assessment.

Studies also reported markedly high OSA burden in special patient populations, although sample sizes were very small. Among pregnant women recruited from an antenatal setting, four out of 10 First Nations Australian pregnant women had OSA (AHI ≥ 5 events/h), compared with 13% (n = 9 out of 70) in non-First Nations Australian Caucasian women [49]. In a study of First Nations Australians with Machado-Joseph disease, recruited from a patient support organization, prevalence was 47% (n = 10 out of 21) [58].

These figures reflect conditional prevalence among patients referred for sleep monitoring, and in special populations, and should not be interpreted as population-level prevalence. Overall, these findings suggest that among Indigenous patients undergoing sleep assessments, OSA is not only highly prevalent, but it is often severe.

#### Audit-based measurement

In contrast to referred clinical samples, studies relying on audit-based diagnostic codes predominantly reported lower recorded rates of OSA among Indigenous patients compared to non-indigenous peers. For example, among U.S. adults hospitalized with SARS-COV-2, 9.3% of American Indian/Alaska Native (AI/AN) patients had an OSA diagnosis, compared to 15.7% of non-AI/AN [59]. Similar patterns were observed in other U.S. hospital cohorts, where recorded OSA rates were lower among Indigenous patients than White patients, but broadly comparable to other minority populations such as African Americans and Hispanics [59, 68].

Comparable patterns were evident in Australian studies. Among remote Australians referred to a specialist respiratory outreach service, 11% of Indigenous patients had OSA recorded as a comorbidity compared with 41% of non-Indigenous patients [43]. In patients with pulmonary hypertension, OSA was recorded in 7% of First Nations Australian patients versus 15% of non-Indigenous patients [53].

These findings suggest that audit-based diagnostic codes may underestimate true prevalence of OSA among Indigenous populations, likely reflecting broader problems of underscreening and systematic access disparities.

### Factors associated with OSA among indigenous and first nations populations

Factors associated with OSA presence and severity are presented in Table 3.

**Table 3:** Factors associated with obstructive sleep apnea among Indigenous and First Nations populations across clinical and community settings

Study ref. (year) country	Indigenous population characteristics & comparator (yes/no)	Sample type; recruitment setting	OSA assessment method & OSA threshold	Primary outcome (Presence/Severity/other)	Key findings
*Indigenous Identity as multivariate predictor*					
Baldwin (1998) [32] New Zealand	Māori, n = 48 (20.6%); 85% Male; *Mdn* Age = 48.5. Comparator(s): Yes	Referred clinical sample. Clinic.	PSG: PSG derived severity markers Clinically Significant OSA: ESS ≥ 12 and A/H index ≥15.	Severity Presence	In multivariate stepwise regressions, being Māori was not a significant independent predictor of any severity marker or presence of OSA. Predictors of OSA not stratified by ethnicity.
Mihaere (2009) [36] New Zealand	Māori, n = 3195(46%); 45% Male; M_age_ = NR. Comparator(s): Yes	Community survey sample; Community-based.	In-home PSG OSA: RDI ≥15	Presence	In a multivariate logistic regression (including fixed ethnicity, sex, age, BMI, neck circumference), being Māori was not an independent predictor of OSA presence. Predictors of OSA not stratified by ethnicity.
Woods (2015) [41] Australia	Aboriginal and Torres Strait Islander Peoples, n = 100(50%); 52% Male; M_age_ = 47.3 (*SD* = 12.6). Comparator(s): Yes	Special population (regional and remote patients with OSA); Clinic/hospital (audit)	PSG: OSA severity	Severity	Using a Mann–Whitney U test, OSA severity did not differ by Indigenous status.
^**^Garg (2020) [46] Australia	“Indigenous Australians”, n = 60(6.3%); % male: NR; Mdn age = 48. Comparator(s): Yes	Referred clinical sample; Clinic (audit).	PSG: OSA: AHI ≥ 5 Positional Sleep Apnea: supine AHI to lateral AHI ratio ≥ 2)	Other	In a multivariate logistic regression of patients with OSA, ethnicity was not a significant multivariate predictor of POSA.
^**^Heraganahally (2022) [57] Australia	“Indigenous Australians”, n = 86 (14.5%) 100% Women; Mdn age = 42.8. Comparator(s): Yes	Referred clinical sample; Clinic (audit).	PSG: OSA: AHI ≥5 Severity: Mild, moderate and severe categories.	Presence Severity	Using logistic regressions (univariate and multivariate), indigenous status was not a significant predictor of OSA presence. Using ordered regression analysis, being Indigenous was a significant univariate, but not multivariate predictor of increased OSA severity. Predictors of OSA not stratified by ethnicity.
*Demographic, anthropometric & geographic factors*					
Heinsberg (2024) [70] Samoa	Samoan adults N = 330(100%); 46.7% Male; M_age_ = 52.9 (SD = 10). Comparator(s): No	Community sample; Community based.	Validated home sleep apnea device (WatchPAT, Itamar): OSA severity: continuous AHI 3%, 4%, and ODI 4%.	Severity	In a multiple linear regression, the following variables predicted at least one OSA severity marker: Higher age, Male sex, Higher BMI, Higher Abdominal-hip, circumference ratio, Census region (Northwest ‘Upolu census region having higher values vs Apia Urban Area census region).
Wu (2024) [72] United States	American Indian participants, N = 123 (100%); 41% Male; M_age_ = 63. Comparator(s): No	Community sample; Cohort study/studies.	Home PSG OSA diagnosis (using REI)	Severity	Multiple logistic regression showed that male sex and BMI were independent predictors of OSA diagnosis; however, asthma was not a statistically significant risk factor for OSA.
Dosman (2022a) [56] Canada	Cree First Nations communities, N = 233 (100%) 42% Male; M_age_ = 38 (*SD* = 15). Comparator(s): No	Community survey sample; Community-based.	Home PSG: OSA severity categories: none/normal—AHI < 5 per hour; mild—AHI ≥ 5, but <15 per hour; moderate to severe –AHI ≥ 15 per hour.	Severity	Multiple ordinal logistic regression, after adjustment, being male, obese, and loud snoring were significant independent predictors of having mild to severe OSA (vs none/normal).
^^Del Brutto (2020) [45] Ecuador	Amerindian N = 167 (100%); 36% Male; M_age_ = 71.7(*SD* = 6.9). Comparator(s): No	Community sample; Community-based.	PSG: OSA: AHI ≥5	Presence	In a multivariate logistic regression model (covariates including age, gender, BMI, blood pressure, fasting glucose, total cholesterol, neck grasp, and neck circumference), only neck circumference was a significant predictor.
^**^Mehra (2020) [48] Australia	Aboriginal Australians^ diagnosed with OSA. N = 297 (100%) 51.8% Male; Mdn age = 47. Comparator(s): No	Referred clinical sample; Clinic (audit).	PSG: Mild OSA: AHI ≥5-230 Severe OSA: AHI >30	Severity	In an ordered logistic regression, stratified by gender, higher odds of severe OSA (vs mild/moderate) were higher neck circumference in females, and higher ESS scores in males.
^**^LaGrappe (2022) [58] Australia	Aboriginal and Torres Strait Islander Australians, N = 24 (100%); 29% Male; M_age_ = 45.6. Comparator(s): No	Special population (Machado-Joseph Disease); Patient support organisation	PSG: OSA: AHI >5/hour Severity: AHI total score	Presence Severity	Univariate linear regressions showed significant positive association between BMI and AHI total score. Univariate logistic regressions showed no significant effect of any parameters on odds of OSA.
*Craniofacial factors*					
Coltman (2000) [33] New Zealand	Māori, n = 49 (47%); 100% Male, M_age_ = 49.6 (*SD* = 11.8) Comparator(s): Yes	Special population (men with moderate OSA); Clinic.	PSG: Severity marker: RDI (events/hr)	Severity	In stepwise multiple regressions, after adjustment for BMI, within the Māori group, significant independent predictors of RDI were nasal aperture width and retrognathic mandible
*Symptoms & PSG derived parameters*					
O’Connor (2003) [34] United States	American Indian, n = 643 (4.9%) 43% Male; M_age_ = 60.9. Comparator(s): Yes	Community survey sample; Cohort study/studies.	In-home PSG OSA: AHI ≥15	Presence	In a multiple logistic regression, self-reported frequent snoring was independently associated with OSA presence in American Indians.
^**^Heraganahally (2020) [47] Australia	Aboriginal and Torres Strait Islander People, n = 403(13%); 50.9% Male; Mdn age = 47.8. Comparator(s): Yes	Referred clinical sample; Clinic (audit).	PSG: None, mild and moderate OSA Severe OSA (AHI > 30)	Severity	In a multivariate logistic regression (covariates included significant univariate predictors), Stage REM percentage and Respiratory Arousal index were independent predictors of severe OSA.
^^Del Brutto (2019) [44] Ecuador	Amerindian N = 201 (100%); 36% Male; M_age_ = 71.2(*SD* = 7.5). Comparator(s): No	Community sample; Community-based.	PSG: Mild OSA: AHI ≥5-14.99 Moderate to Severe OSA: AHI ≥15	Severity	Ordinal logistic regression showed no significant association of Friedman’s tongue position (FTP) and OSA.
*Comorbidities and health consequences*					
^**^Heraganahally (2021) [51] Australia	Aboriginal Australians^ N = 340(100%); 51% Male; Mdn age = 47. Comparator(s): No	Referred clinical sample; Clinic (audit).	PSG: OSA: AHI ≥5	Other	Descriptive findings suggest that OSA and cardiac disease co-occur in 47% of sample, and that patients with OSA had a higher number of comorbidities compared to those without; however, the proportion of cardiac disease among those with and without OSA was not statistically significantly different. Univariate negative binomial regression analyses suggests that OSA severity was not associated with the rate of hospital admissions.
^Ikram (2023) [65] Canada	Cree First Nations communities, N = 588 (100%) 44.22% Male; 54.25% between 18 and 39 years. Comparator(s): No	Community survey sample; Community-based.	Self-reported physician diagnosed sleep apnea	Other	Multivariable logistic regressions showed that OSA was associated with higher odds of vision problems.
^**^Howarth (2023) [64] Australia	Aboriginal Australians”, N = 248 (100%) 48% Male; Mdn age = 46. Comparator(s): No	Referred clinical sample; Clinic (audit).	PSG: OSA: AHI ≥ 5	Other	Multivariate regression showed no significant difference in spirometry parameters among patients with no OSA, and those with mild, moderate, or severe OSA.
Desai (2023) [61] United States	n ~ 667(0.5%); % Male: NR; Age NR. Comparator(s): Yes	Special population (older persons with OSA and prior stroke history); Administrative data (audit).	Diagnostic codes: Identified OSA on National Inpatient Sample system (2019)	Other	Descriptive findings suggest that among those with OSA and prior history of stroke, the rate of subsequent stroke was highest in Asian/Pacific Islanders (6%) and Native Americans (5.6%), followed by white (4.9%), blacks (4.6%), and Hispanics (4.1%).
Desai (2023b) [60] United States	n ~ 4564(0.4%), % Male: NR; Age NR. Comparator(s): Yes	Special population (geriatric patients with OSA); Administrative data (audit).	Diagnostic codes: Identified OSA on National Inpatient Sample system (2018)	Other	In a multivariate analysis, being Native American (compared to White ref) increases the likelihood of Major Adverse Cardiac and Cerebrovascular Events (MACCE) in G-OSA patients, but this finding was not a statistically significant independent risk factor.
Fonseca (2024) [69] United States	American Indians, N = 160 (100%); 30.63% Male; M_age_ = 62.6(SD = 5.4), with 139 participants undergoing PSG. Comparator(s): No	Community sample; Cohort study/studies.	PSG, OSA: Obstructive Apnea Hypopnea Index (all desats) and + (≥4% desats)	Other	In a multiple linear regression, Sleep Latency, but no other PSG derived characteristic, predicted later clinically relevant cognitive impairment, after adjustment for age, BMI, sex, study site, years of education, depression, and the APOE e4 allele.
Karunanayake (2021) [52] Canada	Cree First Nations communities, N = 567(100%); 43.7% Male; M_age_ = 40(*SD* = 15.3). Comparator(s): No	Community survey sample; Community-based.	Self-reported ever diagnosis of sleep apnea	Other	Bivariate associations revealed that sleep apnea was associated with sleep deprivation, but sleep apnea was not retained in multivariate regression models.
^^Del Brutto (2017) [42] Ecuador	Amerindian N = 38 (100%); 44% Male; M_age_ = 74.5(*SD* = 6.8). Comparator(s): No	Community sample; Community-based.	PSG: OSA = AHI ≥10 episodes/hr	Other	Descriptive and Fisher’s exact test showed Abnormal pneumatization of skull bones in 5 out of 15 (33%) Amerindians with OSA, and only 1 out of 23 without (4%).
Perkins (2022) [59] United States	American Indias/Alaska Natives n = 43(46%) 48.8% Male; M_age_ = 51.9(SD = 12.7). Comparator(s): Yes	Special population (COVID-19 patients); Hospital (audit)	Sleep apnea diagnosis via medical chart review and verbal interview	Presence	Using Fisher’s exact test, there was no significant difference in the proportion of patients with comorbid sleep apnea between AI/AN and non-AI/AN groups.
Lin (2024) [71] United States	American Indian/Alaska Native n = 36 158 (n = 12 685 after matching); Male: NR; Age NR. Comparator(s): Yes	Special population (received a SARS-CoV-2test); Hospital (audit)	Diagnostic codes: OSA diagnosed by information from database codes (ICD‐10 = G47.33)	Presence	Using Cox proportional hazards regression, a COVID-19 infection was a risk factor for a “new OSA diagnosis”

Findings are drawn from multivariate analyses where available, univariate results are reported only when multivariate data are absent or provide important context. Associated factors are reported within Indigenous groups only when stratified. AHI = Apnea-Hypopnoea index (events/hour); AI/AN = American Indian/Alaska Natives; ESS = Epworth Sleepiness Scale; FTP = Friedman’s Tongue PositionICU = Intensive Care Unit; MJD = Machado-Joseph Disease; Mdn = Median; M = Mean; N/n – Sample size/subsample size; NR = Not reported; ODI = Oxygen Desaturation Index; PSG = Polysomnography; RDI = Respiratory Event Index; SD = Standard Deviation;

Comparative prevalence data for non-Indigenous or other ethnic groups are provided where available.

Studies marked ^**^originate from the same clinical service; studies marked ^^derive from the same cohort.

#### Demographic factors

Across studies that stratified findings or reported within-group analyses, older age and male sex were commonly associated with increased OSA presence and/or severity. Age showed a positive association with OSA severity in Samona adults [70]. Male sex was an independent predictor of OSA [56, 70, 72].

#### Anthropometric indicators

Body Mass index was the most frequently reported anthropometric predictor of OSA presence/severity [56, 58, 70, 72]. Additional measures included higher abdominal-Hip ratio [70], and neck circumference, which independently predicted OSA [45], with sex-specific patterns observed (higher neck circumference in females and higher Epworth Sleepiness Scale (ESS) in males associated with more severe OSA [48].

#### Craniofacial features

Craniofacial features, including nasal aperture width and retrognathic mandible, were significant predictors of OSA severity, after adjustment for BMI, within the Māori group [33].

#### Symptoms and PSG-derived markers

As expected, snoring was associated with OSA presence [34, 56]. PSG-derived parameters, such as percentage of REM sleep and respiratory arousal index, were independently associated with severe OSA in Aboriginal and Torres Strait Islander patients [47]. Elevated ESS scores were associated with more severe OSA among males in Aboriginal Australian samples [48].

#### Contextual and exposure factors

In the study of Samoan adults, non-urban residence/region was associated with higher AHI and Oxygen Desaturation Index (ODI) values [70]. Among AI/AN individuals, a COVID-19 infection increased the risk of a subsequent new OSA diagnosis [71].

### Comorbidities and health consequences

Several studies examined the relationship between OSA and potential downstream or broader health outcomes of OSA among Indigenous populations. These studies found that OSA had significant associations with the following outcomes: vision problems (severe eyesight problems mentioned by doctor or nurse practitioner) [65], a higher prevalence of subsequent stroke among Asian-Pacific Islanders and Native Americans compared to other ethnicities [61], sleep deprivation [52], abnormal pneumatization of skull bones [42], and a higher number of comorbidities [51]. These relationships likely have complex (and possibly bi-directional) pathophysiological underpinnings, and only the study examining vision problems demonstrated a significant independent relationship after adjusting for key covariates in a multivariate analysis [65]. Some notable non-significant findings included that OSA was not associated with spirometry parameters among Indigenous Australians in multivariate analyses [64], being Native American was not significantly associated with an increase in the likelihood of Major Adverse Cardiac and Cerebrovascular Events (MACCE) in Geriatric OSA patients [60], and OSA was not associated with cognitive performance in American Indian adults 10 years later [69].

#### Summary

Across Indigenous and First Nations populations, similar associated factors to those identified in non-indigenous populations were identified, including age, male sex, BMI, and neck circumference. Symptoms of OSA, including snoring, and key PSG markers were consistently associated with OSA presence/severity, with craniofacial morphology also contributing in Māori men. Evidence for downstream consequences of OSA was mixed.

### Treatment and management of OSA in indigenous and first nations populations

Predictors and outcomes of CPAP treatment acceptability and adherence among Indigenous populations are presented in Table 4.

**Table 4:** Predictors and outcomes of CPAP treatment acceptability and adherence among Indigenous populations

Study name	Sample	Treatment	Measurement of outcome	Key findings	Predictors of outcome (i.e. treatment response or adherence)
Bakker (2011) [38]	Maori (n = 25) vs non-Maori (n = 101); patients >25 years referred for first CPAP treatment	4-week supervised home trial of CPAP (Laboratory titration [n = 93] or titration by auto-PAP device for ≥7 nights at home [n = 33])	CPAP adherence ≥4 hours/ per night during follow up period	Adherence: Maori: 73.1% vs non-Maori: 79.2%. Mdn CPAP use: 5.1 h/night vs non-Maori 5.7 h/night, *p* = .05.	Ethnicity was not a univariate predictor (OR = 0.83, 95% CI: 0.30-2.34); ns in multivariate model. Predictors within Maori only sample not explored.
Bakker (2014) [39]	Maori (n = 5), Pacific Peoples (n = 5), NZ European (n = 8); focus group of patients >25 years referred for first CPAP treatment.	CPAP	CPAP adherence (hours/night) (Mean) Baseline and End-trial ESS (Mean)	Maori: 5.1 h/night, Pacific 6.8 h/night, NZ European 6.7 h/night adherent. ESS ↓: Maori 15 → 5.8, Pacific 13.5 → 5, NZ European 11.5 → 7	Not explored.
Woods (2015) [41]	Aboriginal and Torres Strait Islander (n = 100) vs non-Indigenous (N = 100); confirmed sleep-disordered breathing.	Treatment recommendations: CPAP 69 vs 66 (ns); ENT Referral 3 vs 7 (ns); lifestyle modification 41 vs 14 (*p* < .001); bilevel ventilation 3 vs 1 (ns)	Missed follow-up: % with no review attended post diagnostic study	Missed follow-up: Aboriginal/Torres Strait Islander 38% vs non-non-Indigenous 19% (*p* = .014).	Not explored
Heraganahally (2021) [51]	Aboriginal Australian patients with OSA diagnosis	Treatment recommendation CPAP (n = 197, 76% of sample)	Compliance: medical record entries (subjective usage) + objective CPAP download (when available)	CPAP compliance: 43%	Not explored
Heraganahally (2022) [57]	Australian women who had undergone a diagnostic sleep study	CPAP	Adherence: medical records + objective CPAP data (subjective report if objective unavailable)	Data for CPAP adherence not disaggregated for Aboriginal and/or Torres Strait Islander women.	Being indigenous not a significant predictor; only ESS and severe desaturation associated with adherence. Predictors within Indigenous only sample not explored.
Heraganahally, Ford (2023) [62]	Adult Aboriginal Australian Patients undergoing PSG for clinically suspected sleep disorder with AHI >5 that were recommended CPAP therapy, n = 659	CPAP	Acceptability: CPAP/APAP trial ever recorded Adaptability: CPAP ≥30 days) Adherence: Among adapters, ≥ 4 h/night for ≥70% nights over ≥30 days.	Acceptability: 315/total (49%); adaptability: 196/315 (62%). Non-adapters: CPAP 1-3 days (n = 102/119) Adherence: 67/195 (33%)	Acceptability predictors: ↑ESS, obesity, ↑neck circumference, severe OSA; adaptability predictors: ↑BMI, severe OSA, outer regional residence (vs remote/very remote); adherence predictor: outer regional residence
Locke (2023) [67]	Native Hawaiian/Pacific Islanders undergoing sleep studies in Utah, with PAP machine downloads (n = 91)	CPAP	Usage: % of nights with any PAP machine usage Adherence (≥70% nights used for ≥4 hours/ per night) Median minutes of PAP usage per night	Usage = 47% (range 5-87%] Adherence: 41% Median PAP usage: 204 minutes (range 51–367)	Higher BMI (37% higher odds per 5 kg/m2, 95%CI = 7.7%–74%) and lower AHI (16% higher odds per 10 events/h, 95%CI 1.7%–29%) were independently associated with likelihood of meeting adherence targets.
Lindfield (2023) [66]	Adult Aboriginal Australian patients who underwent diagnostic and in-lab CPAP implementation studies.	CPAP	Treatment response: PSG derived measures Patient-reported sleep	Diagnostic study → On CPAP: Arousal index: 29 → 17/h, sig Total AHI: 48 → 9/h, sig NREM AHI: 47 → 8/h, sig REM AHI: 56 → 8/h, sig Sp02 Nadir: 77% → 85%, sig Patient-reported sleep: “better than normal”: 12% → 54%, sig.	Multivariate: Age ↑: ↑WASO; ↓TST; ↓SE; ↓ Sp02 (NREM & REM) in CPAP vs diagnostic study -BMI ↑: ↓NREM stage 2; ↑REM AHI in CPAP vs diagnostic study -Male sex: ↑REM AHI; ↑TAI; ↓N3% in CPAP vs diagnostic study
Howarth (2023) [64]	Adult Aboriginal and Torres Strait Islander patients who underwent diagnostic PSG and spirometry	CPAP	Adherence: ≥ 4 h/night for ≥70% nights Spirometry categories: Restrictive, obstructive, mixed, normal (no impairment).	Trailed CPAP: 60% CPAP >30 d: 59% Days used (%) = 30.4 (10.0-91.1) Average hrs/night = 4.85 hrs (3.1, 7.0) Days >4 h: 21.5 (3.3–76.7) Adherent: 30%	For users reporting ≥30d CPAP use: those with restrictive spirometric impairment reported ≥4 hr/night on a Mdn of 13% nights; compared to no impairment with a Mdn of 44% of nights. Adherence: Any spirometric impairment 21% vs no impairment, 39%.

Ns = not significant.

#### Interventions to prevent OSA

No studies evaluating interventions to prevent OSA in adult First Nations populations were identified.

#### Treatment strategies

Among studies that investigated OSA treatment, CPAP was the primary treatment strategy [38, 39, 41, 51, 57, 62, 64, 66, 67]. Woods et al. [41] compared treatment recommendations given to Aboriginal and Torres Strait Islander and non-Indigenous patients, finding that CPAP was most common (69/100 vs 66/100 patients, respectively), with a smaller proportion of patients receiving ENT referral (3/100 vs 7/100), lifestyle modification (41/100 vs 14/100, *p* < .001), and bilevel ventilation (3/100 vs 1/100).

#### Effectiveness of CPAP

No randomized controlled trials assessed CPAP effectiveness in adult Indigenous Peoples. Two studies reported pre-to-post CPAP outcomes: In a small qualitative study (n = 5 Māori; baseline AHI events/hour, *M = 93; SD = 45.8*) [39], mean ESS decreased from 15 (SD =8.3) at baseline to 5.8 (SD = 6.4]) at follow-up. Lindfield et al. [66] reported significant improvements in PSG-derived indices among First Nations Australians, with patient-reported sleep quality also improved.

#### Adherence to CPAP

Seven studies reported adherence rates [38, 39, 51, 57, 62, 64, 67] with variation in thresholds used to define it. Rates varied widely: 30% [64], 33% [62], 41% [67], and 43% [51] in Indigenous only samples. Bakker et al [38] found 73.1% of Maori participants were adherent compared to 79.2% of non-Maori participants, with Maori participants showing lower median usage (5.1 vs 5.7 h/night), with ethnicity not a significant univariate or multivariate predictor of adherence. A small qualitative study [39] reported mean CPAP use of 5.1 h/night for Maori participants, 6.8 h/night for Pacific Islanders, and 6.7 h/night for NZ European groups, with a significant difference between Maori and NZ European group.

#### Predictors of adherence

Across studies examining CPAP adherence among Indigenous populations, adherence was associated with clinical severity markers and contextual factors. Predictors of greater adherence included outer regional (vs remote/very remote) residence among Aboriginal Australians [62], higher BMI and lower AHI among Native Hawaiian/Pacific Islander peoples [67], and the absence of spirometric impairment among Aboriginal Australians [64]. These findings suggest that adherence is influenced by a symptom burden, condition severity, and geographic accessibility.

#### Summary

Across included studies, evidence on the treatment and management of OSA in Indigenous peoples is limited and largely observational. CPAP remains the dominant treatment approach. Adherence to CPAP is highly variable across studies. The association between geographic context and CPAP adherence is notable, suggesting that geographic accessibility may independently shape treatment outcomes beyond individual clinical factors, and highlighting the importance of considering geographic context when interpreting adherence patterns in First Nations populations.

### Assessment of study quality

Due to the designs of the studies included in the review, only the observational cohort and cross-sectional study tool from the NIH was utilized. The results of the general quality assessment are reported in Supplementary Materials, Table S3. An additional quality assessment was performed, evaluating adherence to best practice in First Nations research; these results are reported in Supplementary Materials Table S4. These quality evaluations found a preponderance of cross-sectional and cohort studies in the literature addressing OSA in Indigenous peoples, particularly retrospective cohort studies from medical record review. The CREATE evaluation demonstrated that, overwhelmingly, First Nations principles and research practices are not routinely followed in this space.

## Discussion

This review found that the prevalence of OSA among Indigenous populations is highly variable, depending on sampling frame, diagnostic thresholds, and measurement methods. True population-based prevalence data for Indigenous peoples globally remain largely unavailable. The review identified a range of factors associated with OSA in multivariate models, including demographic (male sex, age), anthropometric (BMI, neck circumference), craniofacial, geographic, and symptomatic indicators such as snoring. Evidence on OSA treatment in Indigenous populations is limited: only CPAP has been evaluated, with no OSA preventative interventions or randomized controlled trials (RCTs). CPAP adherence was generally low, and predictors of response remain poorly understood. These findings highlight critical gaps in evidence, underscoring the need for culturally tailored interventions and robust epidemiologic studies.

Over a decade ago, Woods and colleagues [20] first examined OSA in adult Indigenous populations across high-income countries worldwide, identifying a limited number of studies. Woods and colleagues’ review highlighted the lack of research in this area, particularly for First Nations Australians. Our review demonstrates that since then, there has been an increase in research focused on Indigenous populations worldwide, with a growing focus on service access barriers [41], and an increasing number of cohort studies, particularly driven by research on First Nations’ Australian cohorts from studies of referred patients from the Northern Territory, which has the highest proportion of First Nations Australians of any Australian state or territory, with approximately one-quarter of the population identifying as Aboriginal and/or Torres Strait Islander.

### Prevalence of OSA among indigenous and first nations peoples across included studies

Understanding the prevalence of OSA among Indigenous peoples is important to identify unmet health needs and unjust sleep health disparities. True population-based studies of OSA among Indigenous populations are limited, with the exception of Mihaere [36], who provided population-based estimates for New Zealand Māori peoples. Sample-based prevalence studies, as well as available studies drawn from community-based research, suggest that there is a high prevalence of OSA among Indigenous peoples identified in this review; and this prevalence tended to be higher than comparison non-Indigenous populations. Very high prevalence estimates from referred clinical samples reflect prevalence among people already selected for symptoms, clinical suspicion, or with access to diagnostic pathways, not population prevalence. Additionally, where OSA is explored among referred samples, OSA is more severe, in terms of polysomnography (PSG) measured indices, when compared against non-Indigenous comparative groups, perhaps suggesting that these populations are only getting referred and having assessment if they are at the severe end of the OSA spectrum.

Based on the included studies, sample-based prevalence varied based on the measurement used to determine OSA presence. In particular, data based on self-reported physician diagnosis or confirmation of diagnosis via medical records showed lower rates of diagnosis compared to non-Indigenous counterparts and revealed substantially lower prevalence than community samples. Similarly, Indigenous populations show high prevalence of OSA symptomatology despite relatively low rates of physician diagnosis [35]. While care must be taken in generalizing given the differing Indigenous populations represented across studies and variable study designs, when considered together, these findings suggest that OSA is likely underdiagnosed in Indigenous populations. Furthermore, when diagnosis does occur, OSA tends to be more severe (indicating those with more mild disease are not being diagnosed). This suggests that there are inequities in access to sleep study diagnostic services and care, or ineffectiveness of such services in these groups.

The included studies varied in their selection of OSA definition and diagnostic modality, which contributed to the variability in prevalence estimates. Home-based and clinic-based polysomnography, clinical diagnoses, and self-reports differ substantially in their sensitivity and specificity for OSA detection, and have different uptake across populations. These methodological differences substantially limit the comparability of prevalence estimates across studies. Together with the limited evidence from population-based studies, these limitations preclude firm conclusion about the true burden of OSA in Indigenous populations globally.

### Associated factors

First Nations Australians understandings of health, well captured in frameworks such as Social and Emotional Wellbeing (SEWB), highlight that health is shaped by social, historical, and structural determinants rather than individual factors alone [11]. However, previous research has primarily examined individual risk factors for OSA, with limited exploration of social determinants within Indigenous populations. The importance of these factors is demonstrated in other literature, exploring explanations for OSA sleep disparities among minority populations [73]. Increasing recognition of social and environmental determinants of OSA among Indigenous populations require further exploration in order to practically improve the risk factors that lead to OSA; and to develop services which diagnose and manage OSA in an accessible and timely manner.

Our review sought to identify multivariate (where available) risk factors of OSA or OSA severity in Indigenous peoples in studies that stratified finding by race/ethnicity. This review found several demographics (male sex, age), anthropometric (BMI, neck circumference, waist to hip ratio), craniofacial, geographic and symptomatic (e.g. snoring) factors that were associated with OSA diagnosis severity within Indigenous populations. These findings suggest promising targets for consideration in interventions to prevent or manage OSA among Indigenous peoples. However, these risk factors are primarily individual-level factors, with socioenvironmental factors explored less frequently, and thus not identified as commonly associated factors.

We also explored the broader clinical impact of OSA beyond its primary symptoms such as sleep quality and daily functioning. However, as many of these studies were cross sectional, (thereby limiting causal inferences), it is unknown whether OSA may contribute to, result from, or coexist with these other health issues. These findings allude to the complex health burden experienced by Indigenous peoples living with sleep disorders, including OSA. Additional caution must also be considered with the use of screening tools, such as the Epworth Sleepiness Scale, that may not be appropriate for use with some groups of First Nations peoples [74], with suggestion that further validation is required in First Nations peoples [75].

### Management of OSA in indigenous populations

CPAP adherence data can serve as a broad indicator of acceptability of the predominant approach to OSA treatment and management. This review found that adherence rates tended to be lower than 50% across the studied Indigenous populations; and that treatment adherence was lower among Indigenous populations compared to their non-Indigenous comparator populations. While these adherence data provide broad insights into the acceptability of OSA treatment among Indigenous peoples, patient satisfaction data and qualitative studies likely provide more direct insights into barriers and factors that increase acceptability. For example, existing qualitative studies (not examined in this review) that conducted in-depth interviews with First Nations peoples suggest that barriers include a lack of knowledge about OSA and treatment options, a sense of shame, remote living conditions, a lack of follow-up and support, and a lack of cultural safety in interventions [21–24]. Notably, some practical requirements of CPAP therapy pose additional challenges for people facing disadvantage or in remote settings, such as access to a stable sleep environment, housing security, and reliable electricity. These practical constraints may pose a more substantial barrier for CPAP than to alternative treatments (such as pharmaceuticals), highlighting the need to interpret CPAP adherence data within broader social and environmental contexts, rather than attributing differences to individual factors alone. Studies also identify that enablers include engaging elders and Aboriginal Health Practitioners in program design and delivery, incorporating cultural practices alongside standard CPAP treatment, and including community in developing a shared understanding of health risks and benefits of OSA and using treatment [21,24,62,76].

Importantly, no randomized controlled trials or controlled trials have evaluated the effectiveness of interventions aimed at improving OSA treatment outcomes. Additionally, no RCTs or controlled trials were identified that aimed to improve uptake of CPAP or CPAP adherence, using methods such as education, culturally responsive care models, or place-based community interventions. These gaps highlighted in this review underscore an urgent need to develop and evaluate the effectiveness of such culturally-responsive and evidence based interventions.

### Opportunities for diagnosis and management of OSA in indigenous peoples

It is likely that any intervention or service model designed to improve OSA awareness, screening or treatment among Indigenous peoples will need to consider a range of structural, geographic, and service-level factors. Barriers to diagnosis and treatment for First Nations Australian peoples include limited awareness of OSA, limited healthcare access, and challenges accessing diagnostic assessments and treatments in non-metropolitan areas, where people from First Nations communities may be disproportionately represented. The assessment and diagnosis of OSA is typically resource intensive and specialist-driven. Gold standard measurement of OSA is derived from PSG which has been historically conducted in laboratory settings, which requires access to appropriate equipment, technical support, and follow-up care. These are typically concentrated in metropolitan centers. Practical barriers such as the need for travel to metropolitan areas and long wait times can make OSA diagnosis particularly challenging for rural and remote contexts, potentially contributing to delays in diagnosis and engagement with treatment [41]. Future research should aim to disaggregate prevalence and risk factor data by geographic setting to inform more targeted and equitable service planning. Efforts to broaden the feasibility of testing by including at-home sleep studies, or other measurement of OSA-indicators, such as pulse oximetry provide promising options to improve accessibility. Consideration should also be extended into treatment and management practices. Alternative therapies, such as positional therapy or oral appliances, which may provide more feasible and pragmatic pathways to improved outcomes in remote or under-resourced settings where unreliable electricity supply or stable housing is a barrier to CPAP adherence. Future research should evaluate their efficacy, acceptability, and implementation within these contexts*.* As understanding of the physiological mechanisms underlying OSA continues to evolve, endotype-guided management may help identify individuals most likely to benefit from non-CPAP therapies, including positional therapy, oral appliances, weight-loss interventions, or pharmacological approaches. Such strategies may improve treatment effectiveness and adherence while reducing reliance on resource-intensive therapies, particularly in remote and underserved communities where access to specialist sleep services and ongoing CPAP support may be limited. Additionally, there has been minimal research into the design of culturally responsive diagnosis and management approaches, despite promising evidence for “culturally-tailored evidence based interventions” for OSA among racial/ethnic minorities [73]. Implementation of such culturally-tailored approaches may be particularly important for Indigenous populations.

### Quality of included studies

Quality assessment of the included studies demonstrated the literature is predominantly comprised of lower grade evidence, mostly cross-sectional and retrospective cohort studies, typically without evidence of major leadership or input from First Nations communities or researchers. A particular challenge in conducting and interpreting study quality for this, and other systematic reviews is the uncertainty regarding conduct of the research which may not have been reported in the published articles. It is possible that some studies had significant Indigenous and First Nations input, but this was not reported in the article. In using the CREATE tool for assessing adherence to First Nations research best practice, we deliberately opted towards rating criteria as “uncertain” rather than assuming the authors did not follow the criteria when examples were not located in the paper. Nonetheless, it is probably the case that in many of these circumstances, the absence of inclusion of these methods in the paper likely does represent their absence during the performance of the study. There were notable exceptions [55, 56, 65], which provide an example of ethical and genuine First Nations community engagement and the value of reporting the approach to this in scholarly outputs.

### Limitations of the review

Although this systematic review employed a comprehensive search strategy, there were limitations that should be acknowledged and considered in interpretation. The exclusion of abstract-only publications has likely resulted in the omission of relevant data, particularly for Native Hawaiian and Pacific Islander populations, where recent findings have been published in conference abstracts and letters [77–81], but not always in peer-reviewed articles suitable for quality appraisal. Additionally, some studies may have been missed due to keyword choices, as the search focused on English-language terms, potentially excluding relevant research on less commonly identified Indigenous groups. In addition, although this review aimed to be inclusive by exploring Indigenous peoples worldwide, there is considerable heterogeneity between Indigenous peoples worldwide, and within Indigenous and First Nations groups. For example, contextual factors such as access to health services vary substantial for Indigenous and First Nations people living in rural and remote areas compared to urban areas. These differences and nuances are difficult to capture in an overarching review. We suggest that when the literature matures, focused reviews and formal meta-analytic synthesis are warranted for specific groups and subgroups within Indigenous populations.

## Conclusions

With the significant impact of OSA on health, well-being, and broader life outcomes, timely diagnosis, screening, and management are crucial. This is especially important in Indigenous and First Nations communities, where already high rates of chronic health issues are further amplified by undiagnosed or unmanaged OSA. Early identification and treatment can improve sleep, reduce OSA severity, and lessen the downstream human, health, and financial cost of untreated OSA. To achieve this, there is a clear need for culturally responsive community co-designed models of care and locally delivered services (e.g. [25]. Strengthening the role of primary care in OSA management can reduce barriers, enhance service accessibility, and improve community acceptability. The Let’s Yarn About Sleep program [25] has already begun community-led co-design in this space, and the findings from these studies may provide a pathway to inform service delivery innovations in other communities.

## Supplementary Material

Supplementary_File__zpag080

## Data Availability

All data were derived from published studies identified in the review, and no new individual participant data were collected.

## Footnotes

1 Throughout this paper, we use the term Indigenous peoples as an umbrella term for International and cross-jurisdictional discussions. We use the term “First Nations Australian peoples” to refer to Australian Aboriginal and/or Torres Strait Islanders, in accordance with guidance from local First Nations communities in Australia. Where original studies refer to specific groups or use alternative terminology (E.g., First Nations, Aboriginal, Indigenous, or tribal names) we retain those terms when citing or discussing their findings to retain the original authors’ language and context.

## References

[1] Senaratna CV, Perret JL, Lodge CJ, et al. Prevalence of obstructive sleep apnea in the general population: a systematic review. *Sleep Med Rev*. 2017;34:70–81. 10.1016/j.smrv.2016.07.002 27568340

[2] Yeghiazarians Y, Jneid H, Tietjens JR, et al. Obstructive sleep apnea and cardiovascular disease: a scientific statement from the American Heart Association. *Circulation.* 2021;144(3):e56–e67. 10.1161/CIR.0000000000000988 34148375

[3] Kirsch DB . Obstructive sleep apnea. *CONTINUUM: Lifelong Learning in Neurology*. 2020;26(4):908–928. 10.1212/CON.0000000000000885 32756228

[4] Reutrakul S, Mokhlesi B. Obstructive sleep apnea and diabetes: a state of the art review. *Chest.* 2017;152(5):1070–1086. 10.1016/j.chest.2017.05.009 28527878 PMC5812754

[5] Wallace A, Bucks RS. Memory and obstructive sleep apnea: a meta-analysis. *Sleep.* 2013;36(2):203–220. 10.5665/sleep.2374 23372268 PMC3543053

[6] Olaithe M, Bucks RS. Executive dysfunction in OSA before and after treatment: a meta-analysis. *Sleep.* 2013;36(9):1297–1305. 10.5665/sleep.2950 23997362 PMC3738038

[7] Kerner NA, Roose SP. Obstructive sleep apnea is linked to depression and cognitive impairment: evidence and potential mechanisms. *Am J Geriatr Psychiatry*. 2016;24(6):496–508. 10.1016/j.jagp.2016.01.134 27139243 PMC5381386

[8] Borsoi L, Armeni P, Donin G, Costa F, Ferini-Strambi L. The invisible costs of obstructive sleep apnea (OSA): systematic review and cost-of-illness analysis. *PloS One*. 2022;17(5):e0268677. 10.1371/journal.pone.0268677 35594257 PMC9122203

[9] Vanek J, Prasko J, Genzor S, et al. Obstructive sleep apnea, depression and cognitive impairment. *Sleep Med*. 2020;72:50–58. 10.1016/j.sleep.2020.03.017 32544796

[10] Blunden S, Yiallourou S, Fatima Y. Sleep health and its implications in first nation Australians: a systematic review. *The Lancet Regional Health–Western Pacific*. 2022;21:100386. 10.1016/j.lanwpc.2022.100386 35199075 PMC8844889

[11] Australian Government Department of Health . National Aboriginal and Torres Strait Islander Health Plan 2021-2031. Canberra (ACT): Commonwealth of Australia; 2021. Available from: https://www.health.gov.au/resources/publications/national-aboriginal-and-torres-strait-islander-health-plan-2021-2031

[12] Minister of Health . Pae Tū: Hauora Māori Strategy. Wellington (NZ): Ministry of Health; 2023. [Available from: https://www.health.govt.nz/publications/pae-tu-hauora-maori-strategy

[13] Government of Canada . About Indigenous health care 2025. Canada: Government of Canada; 2025. Available from: https://www.sac-isc.gc.ca/eng/1626810177053/1626810219482

[14] Gracey M, King M. Indigenous health part 1: determinants and disease patterns. *The Lancet*. 2009;374(9683):65–75. 10.1016/S0140-6736(09)60914-4 19577695

[15] Blunden S, Fatima Y, Yiallourou S. Sleep health in indigenous Australian children: a systematic review. *Sleep Med*. 2021;80:305–314. 10.1016/j.sleep.2021.01.065 33618099

[16] Marchildon GP, Katapally TR, Beck CA, Abonyi S, Episkenew J, Dosman JA. Exploring policy driven systemic inequities leading to differential access to care among indigenous populations with obstructive sleep apnea in Canada. *Int J Equity Health*. 2015;14(1):148. 10.1186/s12939-015-0279-3 26683058 PMC4683910

[17] Fatima Y, Bucks RS, Mamun AA, et al. Sleep trajectories and mediators of poor sleep: findings from the longitudinal analysis of 41,094 participants of the UK biobank cohort. *Sleep Med*. 2020;76:120–127. 10.1016/j.sleep.2020.10.020 33157426

[18] Fernandez DR, Lee R, Tran N, Jabran DS, King S, McDaid L. Association between poor sleep and mental health issues in indigenous communities across the globe: a systematic review. Sleep. *Advances.* 2024;5(1):zpae028. 10.1093/sleepadvances/zpae028 PMC11078045 38721053

[19] Badri M, Alkhaili M, Aldhaheri H, Yang G, Albahar M, Alrashdi A. From good sleep to health and to quality of life–a path analysis of determinants of sleep quality of working adults in Abu Dhabi. *Sleep Science and Practice*. 2023;7(1):1. 10.1186/s41606-023-00083-3

[20] Woods CE, Usher K, Maguire GP. Obstructive sleep apnoea in adult indigenous populations in high-income countries: an integrative review. *Sleep and Breathing*. 2015;19(1):45–53. 10.1007/s11325-014-1032-7 25084982

[21] Fatima Y, Sullivan D, Bucks R, Von Senden R, Edmed S. Sleep apnoea can be scary. But here’s what happened when first nations people had a say in their own care. *The Conversation*. 2023; Nov 8. Available from: https://theconversation.com/sleep-apnoea-can-be-scary-but-heres-what-happened-when-first-nations-people-had-a-say-in-their-own-care-214641

[22] Woods C, Usher K, Edwards A, Jersmann H, Maguire G. The tyranny of distance–mapping accessibility to polysomnography services across Australia. *Australian Indigenous HealthBulletin*. 2015;15(3):1–10.

[23] Fatima Y, King S, Solomon S, Bucks R, Skinner T. P037 indigenous Australians’ conceptualisation of sleep health differs from western interpretations. *SLEEP Advances*. 2021;2(Supplement_1):A33. 10.1093/sleepadvances/zpab014.085

[24] Woods C, Usher K, Kerr L, Ferns J, Maguire G. editors Barriers and enablers to successful uptake of continuous positive airway pressure (CPAP) treatment for obstructive sleep apnoea for aboriginal and Torres strait islander people. *Journal of Sleep Disorders: Treatment & Care*. 2016;5(3):1000179. SciTechnol.

[25] Fatima Y, Edmed SL, Von Senden R, et al. Obstructive sleep apnea diagnosis and management in first nations communities: protocol for the Let’s yarn about sleep-obstructive sleep Apnea program. Sleep. *Advances.* 2025;6(4):zpaf061. 10.1093/sleepadvances/zpaf061 PMC12510461 41080915

[26] Sutherland K, Lee RW, Cistulli PA. Obesity and craniofacial structure as risk factors for obstructive sleep apnoea: impact of ethnicity. *Respirology.* 2012;17(2):213–222. 10.1111/j.1440-1843.2011.02082.x 21992683

[27] Page MJ, McKenzie JE, Bossuyt PM, et al. The PRISMA 2020 statement: an updated guideline for reporting systematic reviews. *BMJ*. 2021;372:n71 10.1136/bmj.n71 PMC8005924 33782057

[28] Paez A . Gray literature: an important resource in systematic reviews. *J Evid Based Med*. 2017;10(3):233–240. 10.1111/jebm.12266 28857505

[29] Australian Institute of Aboriginal and Torres Strait Islander Studies . AIATSIS Code of Ethics for Aboriginal and Torres Strait Islander Research. Canberra (ACT): AIATSIS; 2020. Available from: https://aiatsis.gov.au/research/ethical-research/code-ethics

[30] Rigney L-I . Internationalization of an indigenous anticolonial cultural critique of research methodologies: a guide to indigenist research methodology and its principles. *Wicazo Sa Review*. 1999;14(2):109–121. 10.2307/1409555

[31] Harfield S, Pearson O, Morey K, et al. Assessing the quality of health research from an indigenous perspective: the aboriginal and Torres Strait islander quality appraisal tool. *BMC Med Res Methodol*. 2020;20(1):79. 10.1186/s12874-020-00959-3 32276606 PMC7147059

[32] Baldwin DR, Kolbe J, Troy K, et al. Comparative clinical and physiological features of Maori, Pacific islanders and Europeans with sleep related breathing disorders. *Respirology.* 1998;3(4):253–260. 10.1111/j.1440-1843.1998.tb00131.x 10201052

[33] Coltman R, Taylor DR, Whyte K, Harkness M. Craniofacial form and obstructive sleep apnea in Polynesian and Caucasian men. *Sleep.* 2000;23(7):943–950. 10.1093/sleep/23.7.1h 11083603

[34] O'Connor GT, Lind BK, Lee ET, et al. Variation in symptoms of sleep-disordered breathing with race and ethnicity: the sleep heart health study. *Sleep.* 2003;26(1):74–79 12627736

[35] Froese CL, Butt A, Mulgrew A, et al. Depression and sleep-related symptoms in an adult, indigenous, north American population. *J Clin Sleep Med*. 2008;4(4):356–361. 10.5664/jcsm.27237 18763428 PMC2542493

[36] Mihaere KM, Harris R, Gander PH, et al. Obstructive sleep apnea in New Zealand adults: prevalence and risk factors among Māori and non-Māori. *Sleep.* 2009;32(7):949–956. 10.1093/sleep/32.7.949 19639758 PMC2706899

[37] Punjabi NM, Caffo BS, Goodwin JL, et al. Sleep-disordered breathing and mortality: a prospective cohort study. *PLoS Med*. 2009;6(8):e1000132. 10.1371/journal.pmed.1000132 19688045 PMC2722083

[38] Bakker JP, O'Keeffe KM, Neill AM, et al. Ethnic disparities in CPAP adherence in New Zealand: Effects of socioeconomic status, health literacy and self-efficacy. Sleep. 2011;34(11):1595–1603. 10.5665/sleep.1404 PMC3198214 22043130

[39] Bakker J, O’Keeffe K, Neill A, Campbell A. Continuous positive airway pressure treatment for obstructive sleep apnoea: Maori, Pacific and New Zealand European experiences. *J Prim Health Care*. 2014;6(3):221–228. 10.1071/HC14221 25194249

[40] Pahwa P, Abonyi S, Karunanayake C, et al. A community-based participatory research methodology to address, redress, and reassess disparities in respiratory health among first nations. *BMC Res Notes*. 2015;8(1):199. 10.1186/s13104-015-1137-5 25981585 PMC4440281

[41] Woods CE, McPherson K, Tikoft E, et al. Sleep disorders in aboriginal and Torres Strait islander people and residents of regional and remote Australia. *J Clin Sleep Med*. 2015;11(11):1263–1271. 10.5664/jcsm.5182 26094934 PMC4623124

[42] Del Brutto MD, O. Obstructive sleep Apnea and aberrant pneumatization of skull bones. *Revista Ecuatoriana de Neurología*. 2017;26(3):191–193

[43] Kruavit A, Fox M, Pearson R, Heraganahally S. Chronic respiratory disease in the regional and remote population of the northern territory top end: a perspective from the specialist respiratory outreach service. *Australian Journal of Rural Health*. 2017;25(5):275–284. 10.1111/ajr.12349 28618108

[44] Del-Brutto OH, Mera RM, Zambrano M, Castillo PR. Lack of association between the Friedman’s tongue position type IV and obstructive sleep Apnea In older adults of Amerindian ancestry. *Revista Ecuatoriana de Neurología*. 2019;28(1):16–20

[45] Del Brutto OH, Mera RM, Recalde BY, Castillo PR. Assessment of neck grasp as a screening tool for identifying obstructive sleep apnea in community-dwelling older adults. *J Prim Care Community Health*. 2020;11:2150132720984421. 10.1177/2150132720984421 33356814 PMC7768834

[46] Garg H, Er XY, Howarth T, Heraganahally SS. Positional sleep apnea among regional and remote Australian population and simulated positional treatment effects. *Nature and science of sleep.* 2020;12:1123–1135. 10.2147/NSS.S286403 PMC7723233 33304112

[47] Heraganahally SS, Kruavit A, Oguoma VM, et al. Sleep apnoea among Australian aboriginal and non-aboriginal patients in the northern territory of Australia—a comparative study. *Sleep.* 2020;43(3):zsz248. 10.1093/sleep/zsz248 31608397

[48] Mehra S, Ghimire RH, Mingi JJ, et al. Gender differences in the clinical and polysomnographic characteristics among Australian aboriginal patients with obstructive sleep apnea. Nature and science of. *Sleep.* 2020;12:593–602. 10.2147/NSS.S258330 PMC7455593 32922104

[49] Redhead K, Walsh J, Galbally M, Newnham JP, Watson SJ, Eastwood P. Obstructive sleep apnea is associated with depressive symptoms in pregnancy. *Sleep.* 2020;43(5):zsz270. 10.1093/sleep/zsz270 31782959

[50] Singh M, Hall KA, Reynolds A, Palmer LJ, Mukherjee S. The relationship of sleep duration with ethnicity and chronic disease in a Canadian general population cohort. *Nature and science of sleep.* 2020;12:239–251. 10.2147/NSS.S226834 PMC7167267 32346318

[51] Heraganahally SS, Rajaratnam B, Silva SA, et al. Obstructive sleep apnoea and cardiac disease among aboriginal patients in the northern territory of Australia. *Heart, Lung and Circulation*. 2021;30(8):1184–1192. 10.1016/j.hlc.2021.01.007 33741255

[52] Karunanayake CP, Fenton M, Skomro R, et al. Sleep deprivation in two Saskatchewan first nation communities: a public health consideration. *Sleep Medicine: X*. 2021;3:100037. 10.1016/j.sleepx.2021.100037 34169273 PMC8220004

[53] Naing P, Playford D, Strange G, et al. Top end pulmonary hypertension study: understanding epidemiology, therapeutic gaps and prognosis in remote Australian setting. *Heart, Lung and Circulation*. 2021;30(4):507–515. 10.1016/j.hlc.2020.08.008 32962944

[54] Sanchez JM, Jolly SE, Dewland TA, et al. Incident strokes among American Indian individuals with atrial fibrillation. *J Am Heart Assoc*. 2021;10(6):e019581. 10.1161/JAHA.120.019581 33653124 PMC8174189

[55] Dosman JA, Karunanayake CP, Fenton M, et al. STOP-Bang score and prediction of severity of obstructive sleep apnea in a first nation community in Saskatchewan. *Canada Clocks & Sleep*. 2022;4(4):535–548. 10.3390/clockssleep4040042 36278535 PMC9624327

[56] Dosman JA, Karunanayake CP, Fenton M, et al. Obesity, sex, snoring and severity of OSA in a first nation community in Saskatchewan. *Canada Clocks & sleep*. 2022;4(1):100–113. 10.3390/clockssleep4010011 35323165 PMC8947446

[57] Heraganahally SS, Zaw KK, Tip S, et al. Obstructive sleep apnoea and adherence to continuous positive airway therapy among Australian women. *Intern Med J*. 2022;52(3):440–450. 10.1111/imj.15076 33012105

[58] LaGrappe D, Massey L, Kruavit A, et al. Sleep disorders among aboriginal Australians with Machado-Joseph disease: quantitative results from a multiple methods study to assess the experience of people living with the disease and their caregivers. *Neurobiology of sleep and circadian rhythms*. 2022;12:100075. 10.1016/j.nbscr.2022.100075 35516836 PMC9062757

[59] Perkins DJ, Yingling AV, Cheng Q, et al. Elevated SARS-CoV-2 in peripheral blood and increased COVID-19 severity in American Indians/Alaska natives. *Exp Biol Med*. 2022;247(14):1253–1263. 10.1177/15353702221091180 PMC9379605 35491994

[60] Desai R, Mellacheruvu SP, Akella SA, et al. Major adverse cardiac and cerebrovascular events in geriatric patients with obstructive sleep apnea: an inpatient sample analysis. *Medical Sciences*. 2023;11(4):69. 10.3390/medsci11040069 37987324 PMC10660682

[61] Desai R, Singh S, Mellacheruvu SP, et al. Recurrent/subsequent stroke and associated outcomes in geriatric patients with OSA and prior stroke events: a retrospective study using the 2019 national inpatient sample. *Journal of Personalized Medicine*. 2023;13(5):782. 10.3390/jpm13050782 37240952 PMC10221714

[62] Heraganahally SS, Howarth TP, Perez AJ, et al. Acceptability, adaptability and adherence to CPAP therapy among aboriginal Australians with OSA-“the A5 study”. *Sleep Med*. 2023;102:147–156. 10.1016/j.sleep.2022.12.024 36652894

[63] Heraganahally SS, Howarth TP, Wirth H, Short T, Benn E. Validity of the new ‘top end sleepiness scale’against the STOP-Bang tool in predicting obstructive sleep apnoea among indigenous Australian adults. *Intern Med J*. 2023;53(3):339–347. 10.1111/imj.15633 34800328

[64] Howarth T, Ben Saad H, Heraganahally SS. The impact of lung function parameters on sleep among aboriginal Australians–a polysomnography and spirometry relationship study. *Nature and science of sleep*. 2023;15:449–464. 10.2147/NSS.S409883 PMC10263013 37323655

[65] Ikram B . Association between sleep disorders and vision problems among First Nations people [master's thesis on the Internet]. Saskatoon (SK): University of Saskatchewan; 2023. Available from: https://hdl.handle.net/10388/14827

[66] Lindfield M, Howarth TP, Perez AJ, et al. Obstructive sleep apnea in aboriginal Australians: polysomnographic outcomes and symptom perception post-continuous positive airway pressure implementation. Sleep. *Advances.* 2023;4(1):zpad015. 10.1093/sleepadvances/zpad015 PMC10108651 37193275

[67] Locke BW, Sundar DJ, Ryujin D. Severity, comorbidities, and adherence to therapy in native Hawaiians/Pacific islanders with obstructive sleep apnea. *J Clin Sleep Med*. 2023;19(5):967–974. 10.5664/jcsm.10472 36727487 PMC10152360

[68] Patel UK, Rao A, Manihani GSD, et al. Prevalence and outcomes of depression, obstructive sleep apnea, and concurrent anxiety (DOCA) in stroke survivors: insights from a nationwide study. *Cureus.* 2023;15(7):e41968. 10.7759/cureus.41968 PMC10427155 37588321

[69] Fonseca LM, Finlay MG, Chaytor NS, et al. Mid-life sleep is associated with cognitive performance later in life in aging American Indians: data from the strong heart study. *Front Aging Neurosci*. 2024;16:1346807. 10.3389/fnagi.2024.1346807 38903901 PMC11188442

[70] Heinsberg LW, Pomer A, Cade BE, et al. Characterization of sleep apnea among a sample of adults from Samoa. *Sleep epidemiology*. 2024;4:100099. 10.1016/j.sleepe.2024.100099 39749350 PMC11694763

[71] Lin WC, Wu MC, Wang YH, Lin CH, Wei JCC. The prevalence of obstructive sleep apnea syndrome after COVID-19 infection. *J Med Virol*. 2024;96(1):e29392. 10.1002/jmv.29392 38235910

[72] Wu H, Rhoades DA, Reese JA, Jones KR. Asthma and obstructive sleep Apnea overlap in a sample of older American Indian adults: the strong heart study. *J Clin Med*. 2024;13(18):5492. 10.3390/jcm13185492 39336979 PMC11432384

[73] Johnson DA, Ohanele C, Alcántara C, Jackson CL. The need for social and environmental determinants of health research to understand and intervene on racial/ethnic disparities in obstructive sleep apnea. *Clin Chest Med*. 2022;43(2):199–216. 10.1016/j.ccm.2022.02.002 35659019 PMC9451370

[74] Benn E, Wirth H, Short T, Howarth T, Heraganahally SS. The top end sleepiness scale (TESS): a new tool to assess subjective daytime sleepiness among indigenous Australian adults. *Nature and science of sleep*. 2021;13:315–328. 10.2147/NSS.S298409 PMC7941568 33707978

[75] Van der Spuy I, Karunanayake CP, Dosman JA, et al. Determinants of excessive daytime sleepiness in two first nation communities. *BMC Pulm Med*. 2017;17(1):192. 10.1186/s12890-017-0536-x 29233159 PMC5726026

[76] Fien S, Dowsett C, Hunter CL, et al. Feasibility, satisfaction, acceptability and safety of telehealth for first nations and culturally and linguistically diverse people: a scoping review. *Public Health*. 2022;207:119–126. 10.1016/j.puhe.2022.04.007 35640452

[77] May J, Khamesra K, Sarta-Moran B, Liss D, Hutchison K. 0669 sleep architecture and obstructive sleep Apnea differences among Pacific islanders: a single-Center study in Saipan. *Sleep.* 2025;48(Supplement_1):A291–A292. 10.1093/sleep/zsaf090.0669

[78] Mettias A, Iwai K, Edwards-Calma K, et al. 0658 exploring drivers of PAP therapy adherence and OSA severity: insights from a sequential analysis in NHPI populations in Hawaii. *Sleep.* 2025;48(Supplement_1):A287–A. 10.1093/sleep/zsaf090.0658

[79] Ryujin D, Sundar K, Gilles A. 404 sleep-disordered breathing In native Hawaiians/Pacific islanders with associated comorbidities and adherence to therapy. *Sleep.* 2021;44(Supplement_2):A160–A161. 10.1093/sleep/zsab072.403

[80] Anderson N, Sasaki T, Nakamura C, et al. 0643 differences in clinical presentation of obstructive sleep Apnea in native Hawaiian and Pacific islander patients. *Sleep.* 2025;48(Supplement_1):A281–A. 10.1093/sleep/zsaf090.0643

[81] Mettias A, Iwai K, Edwards-Calma K, et al. Insights into obstructive sleep apnea severity and treatment adherence in native Hawaiian and Pacific islanders. *Sleep Med*. 2025;131:106504. 10.1016/j.sleep.2025.106504 40239596

