# Supplementary material for: A systematic review of obstructive sleep apnea in Indigenous peoples worldwide
Source: Sleep Adv. 2026 Jul 23;7(3):zpag080. doi: 10.1093/sleepadvances/zpag080 (PMC13533188; doi:10.1093/sleepadvances/zpag080)
Supplement: Supplementary file 1 [file supplementary_file__zpag080.docx]

A Systematic Review of Obstructive Sleep Apnea in Indigenous and First Nations Peoples Worldwide

Shannon Edmed ^a,b^; Daniel P Sullivan ^a,c,e^; Michelle Olaithe^f^; Scott Coussens ^g,h^; Philip Terrill^i^; Dwayne Mann ^i^; Danielle Wilson ^e,i^; Wayne Williams ^e,j^; Danny J. Eckert, ^k^; Sheethal Kalmadka ^a,b,e^; Sylistah Gadam^b^; Yaqoot Fatima^e^

Corresponding Author: Shannon Edmed, Child Health Research Centre, The University of Queensland, Brisbane, Australia: [s.edmed@uq.edu.au](mailto:s.edmed@uq.edu.au)

a Child Health Research Centre, The University of Queensland, Brisbane, Australia

b The Australian Research Council Centre of Excellence for Children and Families over the Life Course, The University of Queensland, Brisbane, QLD, Australia

c School of Applied Psychology, Griffith University, Australia

d Griffith Centre for Mental Health, Griffith University, Australia

e Thompson Institute, University of the Sunshine Coast, Australia

f School of Psychological Science, University of Western Australia, Western Australia

g Adelaide University, Adelaide, South Australia

h Women’s and Children’s Health Network, WCH Campus, North Adelaide, South Australia.

i School of Electrical Engineering and Computer Science, University of Queensland, Brisbane, QLD, Australia

j Toowoomba Regional Clinical Unit, Medical School, Faculty of Health, Medicine and Behavioral Sciences, University of Queensland, Toowoomba, QLD, Australia

k Flinders Health and Medical Research Institute (FHMRI) Sleep Health Flagship Program and Adelaide Institute for Sleep Health, Flinders University, Adelaide, Australia.

**Supplementary Materials A: Search Strings**

**PubMed:**

(aboriginal[tiab] OR aborigine[tiab] OR "first nation*"[tiab] OR "first people*"[tiab] OR indigenous[tiab] OR "native people*"[tiab] OR "native population*"[tiab] OR tribes[tiab] OR "torres strait islander*"[tiab] OR "first australian*"[tiab] OR tiwi[tiab] OR maori[tiab] OR "tangata whenua"[tiab] OR aleut*[tiab] OR amerind*[tiab] OR autocht*[tiab] OR eskimo*[tiab] OR inuit*[tiab] OR innuit*[tiab] OR inuk[tiab] OR "american indian*"[tiab] OR "canadian indian*"[tiab] OR (("united states"[tiab] or america*[tiab] or canad*[tiab] or alaska*[tiab] or siberian[tiab] OR hawaiian[tiab] OR "new zealand*"[tiab]) AND native*[tiab]) OR "Indigenous Peoples"[Mesh] OR "Oceanians"[Mesh]) AND (OSA[tiab] OR OSAS[tiab] OR "sleep apn*"[tiab] OR "snore*"[tiab] OR "snoring"[tiab] OR "Sleep Apnea Syndromes"[Mesh])

**EMBASE (via Elsevier):**

(aboriginal:ti,ab OR aborigine:ti,ab OR 'first nation*':ti,ab OR 'first people*':ti,ab OR indigenous:ti,ab OR 'native people*':ti,ab OR 'native population*':ti,ab OR tribes:ti,ab OR 'torres strait islander*':ti,ab OR 'first australian*':ti,ab OR tiwi:ti,ab OR maori:ti,ab OR 'tangata whenua':ti,ab OR aleut*:ti,ab OR amerind*:ti,ab OR autocht*:ti,ab OR eskimo*:ti,ab OR inuit*:ti,ab OR innuit*:ti,ab OR inuk:ti,ab OR 'american indian*':ti,ab OR 'canadian indian*':ti,ab OR (('united states':ti,ab OR america*:ti,ab OR canad*:ti,ab OR alaska*:ti,ab OR siberian:ti,ab OR hawaiian:ti,ab OR 'new zealand*':ti,ab) AND native*:ti,ab) OR 'indigenous people'/exp OR ‘Oceanic ancestry group’/exp) AND (OSA:ti,ab OR OSAS:ti,ab OR 'sleep apn*':ti,ab OR snore*:ti,ab OR snoring:ti,ab OR 'Sleep Apnea Syndromes'/exp)

**CINAHL:**

((TI aboriginal OR AB aboriginal) OR (TI aborigine OR AB aborigine) OR (TI "first nation*" OR AB "first nation*") OR (TI "first people*" OR AB "first people*") OR (TI indigenous OR AB indigenous) OR (TI "native people*" OR AB "native people*") OR (TI "native population*" OR AB "native population*") OR (TI tribes OR AB tribes) OR (TI "torres strait islander*" OR AB "torres strait islander*") OR (TI "first australian*" OR AB "first australian*") OR (TI tiwi OR AB tiwi) OR (TI maori OR AB maori) OR (TI "tangata whenua" OR AB "tangata whenua") OR (TI aleut* OR AB aleut*) OR (TI amerind* OR AB amerind*) OR (TI autocht* OR AB autocht*) OR (TI eskimo* OR AB eskimo*) OR (TI inuit* OR AB inuit*) OR (TI innuit* OR AB innuit*) OR (TI inuk OR AB inuk) OR (TI "american indian*" OR AB "american indian*") OR (TI "canadian indian*" OR AB "canadian indian*") OR (((TI "united states" OR AB "united states") OR (TI america* OR AB america*) OR (TI canad* OR AB canad*) OR (TI alaska* OR AB alaska*) OR (TI siberian OR AB siberian) OR (TI hawaiian OR AB hawaiian) OR (TI "new zealand*" OR AB "new zealand*")) AND (TI native* OR AB native*)) OR (MH "Indigenous Peoples+")) AND ((TI OSA OR AB OSA) OR (TI OSAS OR AB OSAS) OR (TI "sleep apn*" OR AB "sleep apn*") OR (TI snore* OR AB snore*) OR (TI snoring OR AB snoring) OR (MH "Sleep Apnea Syndromes+")).

**Cochrane CENTRAL:**

(aboriginal:ti,ab OR aborigine:ti,ab OR ("first" NEXT nation*):ti,ab OR ("first" NEXT people*):ti,ab OR indigenous:ti,ab OR ("native" NEXT people*):ti,ab OR ("native" NEXT population*):ti,ab OR tribes:ti,ab OR ("torres strait" NEXT islander*):ti,ab OR ("first" NEXT australian*):ti,ab OR tiwi:ti,ab OR maori:ti,ab OR "tangata whenua":ti,ab OR aleut*:ti,ab OR amerind*:ti,ab OR autocht*:ti,ab OR eskimo*:ti,ab OR inuit*:ti,ab OR innuit*:ti,ab OR inuk:ti,ab OR ("american" NEXT indian*):ti,ab OR ("canadian" NEXT indian*):ti,ab OR (("united states":ti,ab OR america*:ti,ab OR canad*:ti,ab OR alaska*:ti,ab OR siberian:ti,ab OR hawaiian:ti,ab OR ("new" NEXT zealand*):ti,ab) AND native*:ti,ab) OR [mh "Indigenous Peoples"] OR [mh Oceanians]) AND (OSA:ti,ab OR OSAS:ti,ab OR ("sleep" NEXT apn*):ti,ab OR snore*:ti,ab OR snoring:ti,ab OR [mh "Sleep Apnea Syndromes"])

**ATSIHealth:**

****Because ATSIHealth already only includes First Nations health articles, only the sleep apnea terms are needed for this database****

(osa) OR (osas) OR (sleep apn*) OR (snore*) OR (snoring)

**Web of Science:**

((TI=aboriginal OR AB=aboriginal) OR (TI=aborigine OR AB=aborigine) OR (TI="first nation*" OR AB="first nation*") OR (TI="first people*" OR AB="first people*") OR (TI=indigenous OR AB=indigenous) OR (TI="native people*" OR AB="native people*") OR (TI="native population*" OR AB="native population*") OR (TI=tribes OR AB=tribes) OR (TI="torres strait islander*" OR AB="torres strait islander*") OR (TI="first australian*" OR AB="first australian*") OR (TI=tiwi OR AB=tiwi) OR (TI=maori OR AB=maori) OR (TI="tangata whenua" OR AB="tangata whenua") OR (TI=aleut* OR AB=aleut*) OR (TI=amerind* OR AB=amerind*) OR (TI=autocht* OR AB=autocht*) OR (TI=eskimo* OR AB=eskimo*) OR (TI=inuit* OR AB=inuit*) OR (TI=innuit* OR AB=innuit*) OR (TI=inuk OR AB=inuk) OR (TI="american indian*" OR AB="american indian*") OR (TI="canadian indian*" OR AB="canadian indian*") OR (((TI="united states" OR AB="united states") OR (TI=america* OR AB=america*) OR (TI=canad* OR AB=canad*) OR (TI=alaska* OR AB=alaska*) OR (TI=siberian OR AB=siberian) OR (TI=hawaiian OR AB=hawaiian) OR (TI="new zealand*" OR AB="new zealand*")) AND (TI=native* OR AB=native*)) OR ALL="Indigenous Peoples" OR ALL=Oceanians) AND ((TI=OSA OR AB=OSA) OR (TI=OSAS OR AB=OSAS) OR (TI="sleep apn*" OR AB="sleep apn*") OR (TI=snore* OR AB=snore*) OR (TI=snoring OR AB=snoring) OR ALL="Sleep Apnea Syndromes")

**PsycINFO:**

((TI aboriginal OR AB aboriginal) OR (TI aborigine OR AB aborigine) OR (TI "first nation*" OR AB "first nation*") OR (TI "first people*" OR AB "first people*") OR (TI indigenous OR AB indigenous) OR (TI "native people*" OR AB "native people*") OR (TI "native population*" OR AB "native population*") OR (TI tribes OR AB tribes) OR (TI "torres strait islander*" OR AB "torres strait islander*") OR (TI "first australian*" OR AB "first australian*") OR (TI tiwi OR AB tiwi) OR (TI maori OR AB maori) OR (TI "tangata whenua" OR AB "tangata whenua") OR (TI aleut* OR AB aleut*) OR (TI amerind* OR AB amerind*) OR (TI autocht* OR AB autocht*) OR (TI eskimo* OR AB eskimo*) OR (TI inuit* OR AB inuit*) OR (TI innuit* OR AB innuit*) OR (TI inuk OR AB inuk) OR (TI "american indian*" OR AB "american indian*") OR (TI "canadian indian*" OR AB "canadian indian*") OR (((TI "united states" OR AB "united states") OR (TI america* OR AB america*) OR (TI canad* OR AB canad*) OR (TI alaska* OR AB alaska*) OR (TI siberian OR AB siberian) OR (TI hawaiian OR AB hawaiian) OR (TI "new zealand*" OR AB "new zealand*")) AND (TI native* OR AB native*)) OR (DE "Indigenous Populations")) AND
((TI OSA OR AB OSA) OR (TI OSAS OR AB OSAS) OR (TI "sleep apn*" OR AB "sleep apn*") OR (TI snore* OR AB snore*) OR (TI snoring OR AB snoring) OR (DE "Sleep Apnea"))

**Grey Literature:**

**Google scholar:**

*Find articles with at least one of the words*

aboriginal aborigine "first nation*" "first people*" indigenous "native people*" "native population*" tribes "torres strait islander*" "first australian*" tiwi maori "tangata whenua" aleut* amerind* autocht* eskimo* inuit* innuit* inuk "american indian*" "canadian indian*" "united states native" "america* native*" "canad* native*" "alaska* native*" "siberian native*" "hawaiian native*" "new zealand* native*" "indigenous peoples" "oceanians"

*with all of the words*

sleep apn*

**Google:**

*Find articles with at least one of the words*

aboriginal aborigine "first nation*" "first people*" indigenous "native people*" "native population*" tribes "torres strait islander*" "first australian*" tiwi maori "tangata whenua" aleut* amerind* autocht* eskimo* inuit* innuit* inuk "american indian*" "canadian indian*" "united states native" "america* native*" "canad* native*" "alaska* native*" "siberian native*" "hawaiian native*" "new zealand* native*" "indigenous peoples" "oceanians"

*with all of the words*

sleep apn*

*site or domain*

(search 1 - organisations) .org, (search 2 – US Government) .gov, (search 3 – Australian Government) .gov.au, (search 4 – New Zealand Government) .govt.nz, (search 5 – UK Government) .gov.uk, (search 6 – Canadian Government) .gc.ca

*File type*

Adobe Acrobat PDF (.pdf)

**GreyNet**

****Broad search justified due to small database size***.*

”apnea”

“apnoea”

**CADTH Grey Matters**

****Broad search justified due to small database size***.*

“apnea”

“apnoea”

**OpenGrey**

****Broad search justified due to small database size***.*

“apnea”

“apnoea”

**BASE**

****Broad search justified due to small database size***.*

“sleep apn”

**MedNar**

Keywords: sleep, Indigenous

**Supplementary Materials**

**Table S1:** Description of studies included in systematic review

| **Study Name Country** | **Other ethnic/ racial groups included?** | **Indigenous Population** | **Indigenous population description** | **Study design and recruitment setting** | **Relevant Focus** | **Study Aim** |
| --- | --- | --- | --- | --- | --- | --- |
| 1.Baldwin et al. (1998) (32)  New Zealand | Yes | Māori | Māori, n = 48 (20.6%);  85% Male;  *Mdn* Age = 48.5. | Prospective study, recruited from **clinic setting**, 1994-1995. | Associated Factors  Prevalence | Compare Māori, Pacific Islanders and Europeans’ clinical and physiological characteristics; assess race/ethnicity as a predictor of sleep apnea severity. |
| 2.Coltman et al., (2000) (33)  New Zealand | Yes | Māori (Polynesian -New Zealand Māori) | Māori, n = 49 (47%);  100% Male,  M_age_= 49.6 (*SD*=11.8) | Cross-sectional study, recruited from **clinic/hospital setting**. | Associated Factors | Examine craniofacial and anthropometric contributors to OSA within groups of Maori and Caucasian men with moderate OSA. |
| 3.O'Connor (2003) (34)  United States | Yes | American Indian | American Indian, n=643 (4.9%)  43% Male;  M_age_=60.9. | Cross-sectional survey (Sleep Heart Health Study), recruited from **population-based cohort** studies on cardiovascular disease and pulmonary disease. | Associated Factors | Compare self-reported snoring, apnea, and daytime sleepiness between six racial/ethnic groups in the US 40 years of age or older. |
| 4.Froese (2008) (35)  Canada | No | Indigenous North American groups (Gitxsan, Nisga’a and Tsimshian) | Indigenous North American groups, N= 430 (100%);  44% Male;  M_age_= 43.2 (*SD*= 14.3). | Cross-sectional survey study, recruited from **community/non-clinical setting**. | Prevalence | Determine the prevalence of sleep symptoms and their relation to depression in Indigenous North American adults |
| 5.Punjabi (2009) (37)  United States | Yes | Native American | Native American, n= 598 (9.5%);  47% Male;  M_age_=62.9 (*SD*=11). | Prospective cohort study (Sleep Heart Health Study), originally recruited from **population-based cohort** studies on cardiovascular disease and pulmonary disease. | Prevalence | Examine the association between sleep-disordered breathing and all cause mortality in American's 40 years and older |
| 6.Mihaere (2009) (36)  New Zealand | Yes | Māori | Māori, n= 3195(46%);  45% Male;  M_age_ = NR. | Cross-sectional survey and home monitoring observational study, recruited from **community setting**. | Associated Factors  Prevalence | Estimate the prevalence, and identify risk factors, and symptom profiles of OSA amongst Māori and Non-Māori New Zealand adults aged 30-59 years |
| 7.Bakker (2011) (38)  New Zealand | Yes | Māori | Māori, n=25, (19.8%)  80.8% Male;  M_age_ = 45 (*SD*=11.4). | Prospective observational study, recruited from single **sleep clinic**, 2010 | Adherence | Examine the influence of ethnicity on adherence with Continuous positive airway pressure (CPAP) in New Zealand Patients |
| 8.Bakker (2014) (39) New Zealand | Yes | Māori | Māori, n =5, (28%);  60% Male;  M_age_ = 39.3 (*SD*=6.2) | Qualitative Study, recruited from single **sleep clinic**, 2010. | Adherence and follow up | Explore Maori, Pacific and NZ European patients’ experience of CPAP treatment. |
| 9.Woods (2015) (41)  Australia | Yes | Aboriginal and Torres Strait Islander Peoples | Aboriginal and Torres Strait Islander Peoples, n=100(50%);  52% Male;  M_age_= 47.3 (*SD* =12.6). | Retrospective cohort study (**medical records audit**), from hospital-based sleep clinics at study sites of Alice Springs and Cairns. | Associated Factors  Adherence | Compare the level of use of sleeps services and prevalence of clinical factors associated with OSA and management of OSA among Aboriginal and Torres Straight Islander peoples and non-indigenous people living in regional and remote northern and central Australia. |
| 10.Pahwa (2015) (40)  Canada | No | First Nation communities in Saskatchewan | First Nation communities in Saskatchewan, N=874 (100%);  49% male;  M_age_= 35.1(*SD*= 14.3). | Prospective cohort study, recruited from **community** | Prevalence | Evaluate individual and contextual factors related to respiratory health (including OSA) in First Nations Saskatchewan Canadians |
| 11.** Kruavit (2017) (43)  Australia | Yes | “Indigenous Australians” (Aboriginal and Torres Strait Islander People) | “Indigenous Australians”, n= 352 (79.2%);  42.3% Male;  M_age_= 53.8(*SD*= 13.7). | Retrospective cohort study (**medical records audit**), of patients in regional and remote NT Top End referred to a specialist respiratory outreach service. | Prevalence | To study the demographic, rates and types of chronic respiratory conditions in Indigenous and non-Indigenous patients in regional and remote communities of Northern Territory |
| 12.^^Del Brutto (2017) (42)  Ecuador | No | Amerindian | Amerindian N= 38 (100%);  44% Male;  M_age_= 74.5(*SD*= 6.8). | Cross-sectional study, recruited from **community** setting | Associated Factors  Prevalence | Examine associations between abnormal skull bone pneumatization and OSA in Amerindians in rural Ecuador |
| 13.^^Del Brutto (2019) (44)  Ecuador | No | Amerindian | Amerindian N= 201 (100%);  36% Male;  M_age_= 71.2(*SD*= 7.5). | Cross-sectional study, recruited from **community** setting | Associated Factors  Prevalence | Examine the relation between Friedman's tongue position and OSA in Amerindians in rural Ecuador |
| 14.^^Del Brutto (2020) (45)  Ecuador | No | Amerindian | Amerindian N= 167 (100%);  36% Male;  M_age_ = 71.7(*SD*= 6.9). | Cross-sectional study, recruited from **community** setting | Associated Factors  Prevalence | Evaluate the utility of neck grasp in screening for OSA in Amerindians in rural Ecuador |
| 15.Singh (2020) (50) Canada | Yes | “Aboriginal” (eg, First Nations, Métis, Inuit) | “Aboriginal” (eg, First Nations, Métis, Inuit) n= 869 (6.1%);  32% Male;  M_age_= 43.5 (*SD*= 12.3). | Cross-sectional survey, recruited from **community** setting. | Prevalence | Examine ethnic differences in sleep duration in Canadian adults |
| 16.Redhead (2020) (49)  Australia | Yes | Aboriginal and Torres Strait Islander Women ≥26 weeks pregnant | Aboriginal and Torres Strait Islander Women ≥26 weeks pregnant, n=10(8.8%);  100% women;  M_age_ = NR | Cross-sectional study, recruited from Hospital setting in Perth between April 2014 and December 2014. | Prevalence | Examine the association between OSA and depression in pregnant Australian women |
| 17.**Mehra (2020) (48)  Australia | No | Aboriginal Australians only (i.e., not Torres Strait Islander or both Aboriginal and Torres Strait Islander). | Aboriginal Australians^ diagnosed with OSA. N= 297 (100%)  51.8% Male;  Mdn age = 47. | Retrospective cohort study (**medical records audit**), from specialist respiratory outreach service. | Associated Factors  Prevalence | Compare gender differences of clinical and PSG characteristics in Aboriginal Australians with OSA |
| 18.**Heraganahally (2020) (47) Australia | Yes | Aboriginal and Torres Strait Islander People | Aboriginal and Torres Strait Islander People, n= 403(13%);  50.9% Male;  Mdn age =47.8. | Retrospective cohort study (**medical records audit**), recruited from a respiratory and sleep service clinic at the Top End Health Service, Darwin Hospital, Northern Territory between 2011-2015 | Associated Factors  Prevalence | Compare OSA and clinical characteristics between Aboriginal and Torres Strait Islander Australians and non-First Nations Australians |
| 19.**Garg (2020) (46)  Australia | Yes | “Indigenous Australians” (Aboriginal and/or Torres Strait Islander People) | “Indigenous Australians”,  n= 60(6.3%);  % male: NR;  Mdn age = 48. | Retrospective cohort study (**medical records audit**), from hospital-based services from the Top End Health Services, Northern Territory. | Associated Factors  Prevalence | Determine the prevalence of positional OSA in rural and remote Australians |
| 20.Naing (2021) (53)  Australia | Yes | Aboriginal and Torres Strait Islander People | Aboriginal and Torres Strait Islander People, n=791(45%);  % Male: NR;  M_age_ = 48(*SD*= 18). | Retrospective cohort study (**medical records audit**), from hospital/clinic health records in the ‘Top End’, Australia, between 2010 and 2015 (followed until death of 2019). | Prevalence | Examine the prevalence of pulmonary hypertension and contributing comorbidities in remote Australians |
| 21.Karunanayake (2021) (52)  Canada | No | Cree First Nations communities in Saskatchewan | Cree First Nations communities,  N= 567(100%);  43.7% Male;  M_age_ = 40(*SD*= 15.3). | Cross sectional cohort study (from baseline from First Nations Sleep Health Project), recruited from **community**. | Prevalence  Comorbid conditions | Examine the prevalence of sleep deprivation and its contributors in Saskatchewan First Nations Canadians |
| 22.Sanchez (2021) (54)  United States | Yes | American Indian | American Indian,  n= 105822(0.62%);  44.6% Male;  M_age_ = 45.5(SD= 17.8). | Retrospective cohort study (**medical records audit**), identified from audit of hospital databases in California. | Prevalence | Compare the incidence of atrial fibrilation and non hemorhagic stroke in American Indians with other ethnicities |
| 23.**Heraganahally (2021) (51) Australia | No | Aboriginal Australians only ^ | Aboriginal Australians^  N=340(100%);  51% Male;  Mdn age = 47. | Retrospective cohort study (**medical records audi**t), identified from a respiratory and sleep service clinic at the Top End Health Service, Darwin Hospital, Northern Territory between 2011-2015 | Prevalence  Adherence  Comorbid conditions | Explore the presence of cardiovascular comorbidities in Aboriginal Australians with OSA |
| 25.Perkins (2022) (59)  United States | Yes | American Indians/Alaska Natives | American Indians/Alaska Natives  n= 43(46%)  48.8% Male;  M_age_= 51.9(SD= 12.7). | Prospective observational study, **recruited from ICU and non-ICU settings** in the University of New Mexico Hospital October 2020. | Prevalence | Examine the clinical and comorbidity predictors of COVID-19 severity in American Indians/Alaskan Natives |
| 26.**LaGrappe (2022) (58) Australia | No | Aboriginal and Torres Strait Islander Australians | Aboriginal and Torres Strait Islander Australians, N= 24 (100%);  29% Male;  M_age_ = 45.6. | Mixed-methods prospective study, with participants with Machado-Joseph Disease (MJD) recruited by convenience sampling from **MJD Foundation clients.** | Associated Factors  Prevalence | Examine the frequency of sleep disorders in Aboriginal Australians with Machado-Joseph Disease |
| 27.**Heraganahally (2022) (57) Australia | Yes | “Indigenous” (Aboriginal and/or Torres Strait Islander People) | “Indigenous Australians”,  n= 86 (14.5%)  100% Women;  Mdn age =42.8. | Retrospective cohort study (**medical records audit**), identified from a respiratory and sleep service clinic at the Top End Health Service, Darwin Hospital, Northern Territory between 2014-2015 | Associated Factors  Prevalence  Adherence | Examine the predictors of CPAP adherence in Australian women with OSA |
| 28.Dosman (2022a)  (56)  Canada | No | Cree First Nations communities in Saskatchewan | Cree First Nations communities, N= 233(100%)  42% Male;  M_age_ = 38(*SD*= 15). | Cross-sectional study from baseline data from the First Nations Sleep Health Project study, identified from a **community setting** between 2018 and 2019. | Associated Factors  Prevalence | Examine the frequency of OSA and its predictors in First Nations Saskatchewan Canadian communities |
| 29.Dosman (2022b)  (55)  Canada | No | Cree First Nations communities in Saskatchewan | Cree First Nations communities, N= 233 (100%)  (215 available for analysis)  41% Male;  M_age_ = 37.7 (*SD*= 14.9). | Cross-sectional study from baseline data from the First Nations Sleep Health Project study, identified from a **community setting** between 2018 and 2019. | Prevalence | Validate the utility of the STOP-BANG score in predicting OSA severity in First Nations Saskatchewan Canadians |
| 30.^Ikram (2023) (65)  Canada | No | Cree First Nations communities in Saskatchewan | Cree First Nations communities, N= 588 (100%)  44.22% Male;  54.25% between 18-39 years. | Cross-sectional study from baseline data from "Assess, Redress, Re-assess: Addressing Disparities in Sleep Health among First Nations People”, identified from **community settings**. | Prevalence  Comorbid conditions | Examine the association between sleep and vision problems in First Nations Saskatchewan Canadians |
| 31.Patel (2023) (68) United States | Yes | Native Americans | Native Americans N=26333 (0.5%)  %male =NR  Age =NR | Retrospective cohort study (**medical records audit**), from the National Inpatient sample from 2003-2017. | Prevalence | Examine the association between depression, OSA, and anxiety in US stroke patients |
| 32.**Heraganahally, Ford (2023) (62) Australia | No | “Aboriginal Australians” (Aboriginal and/or Torres Strait Islander People) | “Aboriginal Australians” (Aboriginal and/or Torres Strait Islander People), N=730(100%), 649 with OSA (53% Male;  Mdn age = 47.7. | Retrospective cohort study (**medical records audit**), identified from a respiratory and sleep service clinic at the Top End Health Service, Darwin Hospital, Northern Territory between 2011-2020. | Prevalence  Adherence | Examine the relation between clinical, demographic, and PSG predictors and CPAP adherence for OSA in First Nations Australians |
| 33.Locke (2023) (67)  United States | No | Native Hawaiians/Pacific Islanders | Native Hawaiians/Pacific Islanders, N= 141 (100%)  68.7% Male;  M_age_ = 46(*SD*=13). | Retrospective cohort study (**medical records/administrative data audit**), recruited from the University of Utah sleep program from 2014-2021. | Prevalence  Adherence | Examine the association between OSA, comorbidities, and CPAP adherence in Native Hawaiian/Pacific Islanders |
| 34.**Lindfield (2023) (66) Australia | No | “Aboriginal Australians” | “Aboriginal Australians” N= 149 (100%)  54% Male;  Mdn age = 48.89. | Retrospective cohort study (**medical records audit**), recruited from services within the Top End Health Service region between 2012-2020 | Prevalence  Treatment effectiveness | Examine the effectiveness of CPAP on subjective and polysomnographic outcomes in Aboriginal Australians |
| 35.**Howarth (2023) (64) Australia | No | “Aboriginal Australians” (Aboriginal and Torres Strait Islander People) | Aboriginal Australians”, N= 248 (100%)  48% Male;  Mdn age = 46. | Retrospective cohort study (**medical records audit)**, recruited from services within Hospitals within the Top End Health Service region between 2012-2020 | Prevalence  Adherence  Comorbid conditions | Examine the effect of lung function on PSG parameters in Aboriginal Australians |
| 36.**Heraganahally, Benn(2023) (63) Australia | No | “Indigenous Australians” (Aboriginal and Torres Strait Islander People) | “Indigenous Australians” N=80 (100%);  51% Male;  M_age_ = 45.1(SD=11.54) | Prospective study, with participants recruited from **services within Hospitals** within the Top End Health Service region between 2017 and 2020 | Prevalence | Validate the utility of the Top End Sleepiness Scale for screening OSA in Indigenous Australian adults |
| 37. Desai (2023) (61) United States | Yes | Native Americans | n ~667(0.5%);  % Male: NR;  Age NR. | Retrospective cohort study (**medical records/administrative data audit**), from the National Inpatient Sample (2019) | Associated Factors | Examine the relationship between recurrent stroke in older persons with OSA and prior stroke history |
| 38.Desai (2023b)(60)  United States | Yes | Native Americans | n~4564(0.4%), % Male: NR;  Age NR. | Retrospective cohort study (**medical records/administrative data audit**), from the National Inpatient Sample (2018) | Associated Factors | Explore burden and predictors of major cardiac and cerebrovascular events in geriatric patients with OSA. |
| 39.Fonseca (2024) (69)  United States | No | American Indians | American Indians,  N= 160 (100%);  30.63% Male;  M_age_ = 62.6(SD= 5.4),  with 139 participants undergoing PSG. | Secondary analyses of overlapping participants in two ancillary studies of the Strong Heart Study: Sleep Heart Health Study (2001-2003) and the Cerebrovascular Disease and its Consequences in American Indians study (2010-2013), **community recruitment** | Prevalence  Associated Factors | Examine associations between sleep characteristics and subsequent cognitive performance in aging American Indians. |
| 40.Heinsberg (2024) (70)  Samoa | No | Samoan adults | Samoan adults  N= 330(100%);  46.7% Male;  M_age_= 52.9 (SD= 10). | Secondary analysis of data collected via the Soifua Manuia (“Good Health” study of Samoan adults (2017-2019). **Community-based recruitment**. | Prevalence  Associated Factors | Examine sample prevalence and characteristics of sleep apnea using a home sleep apnea device and explore factors influencing sleep health in Samoan setting. |
| 41.Lin (2024) (71) United States | Yes | American Indian/Alaska Native | American Indian/Alaska Native  n = 36158 (n= 12685 after matching);  Male: NR;  Age NR. | Secondary analysis of electronic **health record database** (TriNetx Network database). | Associated Factors. | Explore prevalence of new OSA in COVID-19 survivors. |
| Wu (2024) (72) United States | No | American Indian | American Indian participants,  N= 123 (100%);  41% Male;  M_age_=63. | Secondary analyses of overlapping participants in two ancillary studies of the Strong Heart Study: Sleep Heart Health Study and the Asthma-Sub-study. **Community based recruitment.** | Prevalence  Associated Factors | Explore association between asthma and OSA in American Indian communities. |

^Notes. Prevalence estimates reflect the proportion of participants meeting study-specific diagnostic criteria; ESS = Epworth Sleepiness Scale; MJD = Machado-Joseph Disease; Mdn = Median; M = Mean; N/n – Sample size/subsample size; NR = Not reported; SD = Standard Deviation;^

^Comparative prevalence data for non-Indigenous or other ethnic groups are provided where available.^

^Studies marked **originate from the same clinical service;^

**Table S2:** Prevalence of Obstructive Sleep Apnea Symptoms among Indigenous and First Nations populations across diverse settings and measurement methods

| **Study Name (Date), Country (ref).** | **Indigenous population description; n; % male; Age.** | **Study design, recruitment setting** | **Type of OSA symptom** | **Findings:**  **(% of sample that endorsed symptom)** |
| --- | --- | --- | --- | --- |
| Froese (2008), Canada (35) | Indigenous North American groups, N= 430 (100%);  44% Male;  M_age_= 43.2 (*SD*= 14.3). | Community-based recruitment; non-clinical setting; survey conducted exclusively among Indigenous North Americans. | 1. Witnessed Apneas 2. Snoring | Witnessed Apneas: 7.6%  Snoring: 36% |
| Mihaere (2009), New Zealand (36) | Māori, n= 3195(46%);  45% Male;  M_age_ = NR.  Subsample with overnight monitoring (n = 169) | Community-based recruitment; non-clinical setting; monitoring conducted in Maori and non-Maori participants. | 1. Snoring 2. Witnessed Apneas 3. ESS >10 | Snoring: 16.2%  Witnessed Apneas: 30.3%  ESS > 10: 24.6%  *Others*  Non-Maori: Snoring =10.1%, Witnessed Apneas =18.3%, ESS >10 = 15.6% |
| Woods (2015), Australia (41) | Aboriginal and Torres Strait Islander Peoples, n=100(50%);  52% Male;  M_age_= 47.3 (*SD* =12.6). | Hospital/clinic-based medical records audit; Australians (including First Nations) diagnosed with sleep related breathing disorders. | 1. Snoring 2. Witnessed Apneas 3. ESS ≥ 10 | Snoring: 80%  Witnessed Apneas: 49%  ESS ≥ 10: 92%  *Others*  Non-Indigenous Australians: Snoring = 87%, Witnessed Apneas = 58%, ESS ≥ 10 = 97% |
| Pahwa (2015), Canada (40) | First Nation communities in Saskatchewan, N=874 (100%);  49% male;  M_age_= 35.1(*SD*= 14.3). | Community-based recruitment; non-clinical setting; survey conducted exclusively among First Nation communities. | 1. ESS >10 (valid responses) | ESS >10: 10.9% |
| **LaGrappe (2022), Australia (58) | Aboriginal and Torres Strait Islander Australians, N= 24 (100%);  29% Male;  M_age_ = 45.6 | Convenience sampling from MJD Foundation clients; exclusively with First Nations Australians with Machado-Joseph Disease (MJD). | Self-report questionnaire:   1. STOP Bang Low Risk (0-2) 2. Stop Bang Mod Risk (3-5) 3. Stop Bang High Risk (>5) | Low Risk: 50%  Mod Risk: 29%  High Risk: 21% |
| Dosman (2022b), Canada (55) | Cree First Nations communities, N= 233 (100%)  (215 available for analysis)  41% Male;  M_age_ = 37.7 (*SD*= 14.9) | Community-based recruitment; non-clinical setting; monitoring conducted exclusively among First Nation communities. | Questionnaires   1. Loud snoring 2. Observed Apnea 3. Tired/Sleepy 4. Stop Bang ≥5 | Loud snoring: 28.8%  Observed Apnea: 20.5%  Tired/Sleepy: 67.4%  Stop Bang ≥5: 9.8% |
| Ikram (2023), Canada (65) | Cree First Nations communities, N= 588 (100%)  44.22% Male;  54.25% between 18-39 age group | Community-based recruitment; non-clinical setting; survey conducted exclusively among First Nation communities. | Questionnaires   1. Stop Bang ≥ 3 2. ESS>10 | Stop Bang ≥ 3: 43%  ESS>10: 18% |
| Locke (2023), United States (67) | Native Hawaiians/Pacific Islanders, N= 141 (100%)  68.7% Male;  M_age_ = 46(SD=13) | Medical records and administrative data audit from University of Utah Sleep Program. Native Hawaiians/Pacific Islanders with OSA. | 1. ESS>10 | ESS>10: 62% |
| **Heraganahally, Benn(2023), Australia (63) | “Indigenous Australians” N=80 (100%);  51% Male;  M_age_ = 45.1(SD=11.54) | Sleep and respiratory clinic-based recruitment; referred for testing; exclusively among Indigenous Australians. | 1. Stop Bang ≥ 5 | Stop Bang ≥ 5 49% |

^Notes. Prevalence estimates reflect the proportion of participants meeting study-specific diagnostic criteria; ESS = Epworth Sleepiness Scale; MJD = Machado-Joseph Disease; Mdn = Median; M = Mean; N/n – Sample size/subsample size; NR = Not reported; SD = Standard Deviation;^

^Comparative prevalence data for non-Indigenous or other ethnic groups are provided where available.^

^Studies marked **originate from the same clinical service;^

Table S3: National Institutes of Health Quality Assessment for Observational Cohort and Cross-Sectional Studies

| **Study Name** | Clear research aim | Clear study population | ≥50% eligible persons participated | Same population/uniform selection criteria | Power analysis/Sample size justification | Exposure measured prior to outcomes | Sufficient timeframe | Exposure levels measured | Exposure measures valid and consistently applied | Exposure assessed more than once over time | Outcome measures valid and consistently applied | Assessors blinded | ≤20% loss to follow-up | Key confounders measured and adjusted for |
| --- | --- | --- | --- | --- | --- | --- | --- | --- | --- | --- | --- | --- | --- | --- |
| Baldwin et al. (1998) (32) | Y | Y | Y | Y | NR | NA | NA | NR | Y | N | Y | Y | NA | Y |
| Coltman et al., (2000) (33) | Y | Y | Y | Y | Y | Y | Y | Y | Y | N | Y | NR | NA | Y |
| O'Connor (2003) (34) | Y | Y | N | Y | NR | CD | NA | Y | Y | N | Y | NR | NA | Y |
| Froese (2008) (35) | Y | Y | Y | Y | NR | NA | NA | Y | Y | N | Y | NR | NA | Y |
| Punjabi (2009) (37) | Y | Y | Y | Y | NR | Y | Y | Y | Y | Y | Y | NR | Y | Y |
| Mihaere (2009) (36) | Y | Y | Y | Y | NR | NA | NA | NA | Y | N | Y | Y | NA | Y |
| Bakker (2011) (38) | Y | Y | Y | Y | NR | Y | Y | NA | Y | Y | Y | NR | Y | Y |
| Bakker (2014) (39) | Y | Y | N | Y | Y | CD | CD | N | Y | Y | Y | NR | CD | Y |
| Woods (2015) (41) | Y | Y | Y | Y | NR | Y | NA | NA | Y | N | Y | NR | NA | Y |
| Pahwa (2015) (40) | Y | Y | Y | Y | Y | Y | Y | Y | Y | N | Y | NR | Y | Y |
| ** Kruavit (2017) (43) | Y | Y | NA | Y | NR | N | Y | Y | Y | N | Y | NR | NA | Y |
| ^^Del Brutto (2017) (42) | Y | Y | Y | Y | NR | Y | Y | Y | Y | N | Y | Y | Y | N |
| ^^Del Brutto (2019) (44) | Y | Y | N | Y | NR | Y | Y | Y | Y | N | Y | Y | NA | Y |
| ^^Del Brutto (2020) (45) | Y | Y | Y | Y | NR | Y | Y | Y | Y | N | Y | Y | NA | Y |
| Singh (2020) (50) | Y | Y | Y | Y | NR | N | NA | Y | Y | N | Y | NR | NA | Y |
| Redhead (2020) (49) | Y | Y | Y | Y | NR | N | Y | Y | Y | Y | Y | Y | NA | Y |
| **Mehra (2020) (48) | Y | Y | NA | Y | NR | Y | Y | Y | Y | N | Y | Y | Y | Y |
| **Heraganahally (2020) (47) | Y | Y | NA | Y | Y | N | NA | NA | Y | N | Y | NA | NA | Y |
| **Garg (2020) (46) | Y | Y | Y | Y | Y | N | NA | Y | Y | N | Y | NA | NA | Y |
| Naing (2021) (53) | Y | Y | NA | Y | N | Y | NA | Y | Y | N | Y | NA | NA | Y |
| Karunanayake (2021) (52) | Y | Y | Y | Y | Y | N | NA | Y | Y | Y | Y | NR | NA | Y |
| Sanchez (2021) (54) | Y | Y | NA | Y | Y | Y | Y | Y | Y | N | Y | NA | NA | Y |
| **Heraganahally (2021) (51) | Y | Y | Y | Y | Y | N | NA | Y | Y | N | Y | NA | NA | Y |
| Perkins (2022) (59) | Y | Y | Y | Y | NR | Y | CD | Y | Y | Y | Y | NR | Y | Y |
| **LaGrappe (2022) (58) | Y | Y | Y | Y | Y | Y | NA | Y | Y | N | Y | NR | Y | Y |
| **Heraganahally (2022) (57) | Y | Y | Y | Y | Y | Y | Y | Y | Y | N | Y | NA | NA | Y |
| Dosman (2022a)  Obesity (56) | Y | Y | CD | Y | NR | CD | Y | Y | Y | N | Y | NR | NA | Y |
| Dosman (2022b)  STOP-Bang (55) | Y | Y | Y | Y | NR | CD | Y | Y | Y | N | Y | NR | NA | Y |
| ^Ikram (2023) (65) | Y | Y | CD | Y | CD | CD | Y | Y | Y | N | Y | NR | NA | Y |
| Patel (2023) (68) | Y | Y | NA | Y | Y | N | Y | Y | Y | N | Y | NA | NA | Y |
| **Heraganahally, Ford (2023) (62) | Y | Y | Y | Y | Y | Y | Y | Y | Y | N | Y | NR | NA | Y |
| Locke (2023) (67) | Y | Y | NA | Y | Y | Y | Y | Y | Y | N | Y | NR | NA | Y |
| **Lindfield (2023) (66) | Y | Y | Y | Y | Y | Y | Y | Y | Y | N | Y | NR | NA | Y |
| **Howarth (2023) (64) | Y | Y | Y | Y | Y | CD | Y | Y | Y | N | Y | NR | NA | Y |
| **Heraganahally, Benn(2023) (63) | Y | Y | CD | Y | NR | N | Y | Y | Y | N | Y | NR | NR | N |
| Desai (2023) (61) | Y | Y | Y | Y | Y | Y | CD | Y | Y | N | Y | NR | NA | Y |
| Desai (2023b)(60) | Y | Y | NA | Y | NR | Y | NA | N | Y | N | Y | NA | NA | Y |
| Fonseca (2024) (69) | Y | Y | Y | Y | NR | Y | Y | Y | NA | N | Y | NA | CD | Y |
| Heinsberg (2024) (70) | Y | Y | Y | Y | NR | N | Y | Y | Y | N | NR | NA | Y | Y |
| Lin (2024) (71) | Y | Y | NA | Y | NR | NA | Y | NA | Y | Y | Y | NA | CD | Y |
| Wu (2024) (72) | Y | Y | NA | Y | NR | NA | Y | Y | Y | N | Y | NA | CD | Y |

^Abbreviations: Y – Yes, N – No, CD – Cannot determine, NR – Not reported, NA – Not applicable^

Table S4: CREATE Quality Assessment of First Nations Research

| **Study Name** | Responds to community need | Inclusive community consultation | First nations research leadership | First Nations governance | Local community protocols followed | Negotiated rights to existing First Nations I/CP | Negotiated rights to I/CP created through the research | First Nations control over research materials | Guided by First Nations research paradigms | Strengths based approach used | Researchers planned to translate findings to policy/practice | Research benefitted First Nations communities | Conducted First nations capacity strengthening | All involved in the research had opportunities to learn from each other |
| --- | --- | --- | --- | --- | --- | --- | --- | --- | --- | --- | --- | --- | --- | --- |
| Baldwin et al. (1998) (32) | U | U | U | U | U | U | U | U | U | U | Y | U | U | U |
| Coltman et al., (2000) (33) | U | U | U | U | U | U | U | U | U | U | U | U | U | U |
| O'Connor (2003) (34) | U | U | U | U | U | U | U | U | U | U | U | U | U | U |
| Froese (2008) (35) | U | U | U | U | U | U | U | U | U | U | U | Y | Y | U |
| Punjabi (2009) (37) | U | U | U | U | U | U | U | U | U | U | U | U | U | U |
| Mihaere (2009) (36) | U | U | U | U | U | U | U | U | U | U | U | U | U | U |
| Bakker (2011) (38) | U | U | U | U | U | U | U | U | U | U | Y | U | U | U |
| Bakker (2014) (39) | U | U | U | U | Y | U | U | U | U | U | U | U | U | U |
| Woods (2015) (41) | U | U | U | U | U | U | U | U | U | U | U | U | U | U |
| Pahwa (2015) (40) | Y | Y | U | U | U | U | Y | U | U | Y | U | U | U | U |
| ** Kruavit (2017) (43) | U | U | U | U | U | U | U | U | U | U | U | U | U | U |
| ^^Del Brutto (2017) (42) | U | Y | U | U | U | U | U | U | U | U | U | U | U | U |
| ^^Del Brutto (2019) (44) | U | Y | U | U | U | U | U | U | U | U | U | U | U | U |
| ^^Del Brutto (2020) (45) | U | Y | U | U | U | U | U | U | U | U | U | U | U | U |
| Singh (2020) (50) | U | U | U | U | U | U | U | U | U | U | U | U | U | U |
| Redhead (2020) (49) | U | U | U | U | U | U | U | U | U | U | U | U | U | U |
| **Mehra (2020) (48) | U | U | U | U | U | U | U | U | U | U | U | U | U | U |
| **Heraganahally (2020) (47) | U | U | U | U | U | U | U | U | U | U | U | U | U | U |
| **Garg (2020) (46) | U | U | U | U | U | U | U | U | U | U | U | U | U | U |
| Naing (2021) (53) | U | U | U | U | U | U | U | U | U | U | U | U | U | U |
| Karunanayake (2021) (52) | U | U | U | U | Y | U | U | U | U | U | U | U | U | U |
| Sanchez (2021) (54) | U | U | U | U | U | U | U | U | U | U | U | U | U | U |
| **Heraganahally (2021) (51) | U | U | U | U | U | U | U | U | U | U | U | U | U | U |
| Perkins (2022) (59) | U | U | U | U | U | U | U | U | U | U | U | U | U | U |
| **LaGrappe (2022) (58) | U | U | U | U | U | U | U | U | Y | U | U | U | U | U |
| **Heraganahally (2022) (57) | U | U | U | U | U | U | U | U | U | U | U | U | U | U |
| Dosman (2022a)  Obesity (56) | Y | Y | Y | Y | Y | U | Y | Y | Y | U | U | Y | Y | Y |
| Dosman (2022b)  STOP-Bang (55) | Y | Y | Y | Y | Y | U | Y | Y | Y | U | U | Y | Y | Y |
| ^Ikram (2023) (65) | Y | Y | Y | Y | Y | U | Y | Y | Y | U | U | Y | Y | Y |
| Patel (2023) (68) | U | U | U | U | U | U | U | U | U | U | U | U | U | U |
| **Heraganahally, Ford (2023) (62) | U | U | Y | U | U | U | U | U | U | U | U | U | U | U |
| Locke (2023) (67) | U | U | U | U | U | U | U | U | U | U | U | U | U | U |
| **Lindfield (2023) (66) | U | U | U | U | U | U | U | U | U | U | U | U | U | U |
| **Howarth (2023) (64) | U | U | U | U | U | U | U | U | U | U | U | U | U | U |
| **Heraganahally, Benn(2023) (63) | U | U | U | U | U | U | U | U | U | U | Y | U | U | U |
| Desai (2023) (61) | U | U | U | U | U | U | U | U | U | U | U | U | U | U |
| Desai (2023b)(60) | U | U | U | U | U | U | U | U | U | U | U | U | U | U |
| Fonseca (2024) (69) | U | U | U | U | U | U | U | U | U | U | U | U | U | U |
| Heinsberg (2024) (70) | U | U | U | U | U | U | U | U | U | U | U | U | U | U |
| Lin (2024) (71) | U | U | U | U | U | U | U | U | U | U | U | U | U | U |
| Wu (2024) (72) | U | U | U | U | U | U | U | U | U | U | U | U | U | U |

^Abbreviations: I/CP – intellectual and cultural property; U = Uncertain; Y = Y^
